# Proteomic analysis of exosomes from *Brucella abortus*-infected macrophages reveals possible mechanisms of immune evasion and host modulation

**DOI:** 10.3389/fimmu.2025.1685245

**Published:** 2025-10-30

**Authors:** Francisco Álvarez, Vicente Arriagada, María Jesús Aburto, Ítalo Ferrari, Ilse Alvarado, José Barrales, Roberto Vidal, Felipe del Canto, Leonardo A. Gómez, Ángel A. Oñate

**Affiliations:** ^1^ Laboratory of Molecular Immunology, Department of Microbiology, Faculty of Biological Sciences, University of Concepción, Concepción, Chile; ^2^ Interdisciplinary Nucleus of Microbiology, Institute of Biomedical Sciences (ICBM), Faculty of Medicine, Universidad de Chile, Santiago, Chile

**Keywords:** Brucella abortus 2308, exosomes, extracellular vesicles, proteomic analysis, host-pathogen interaction, immune evasion, bacterial Proteins, bacterial persistence

## Abstract

**Introduction:**

*Brucella abortus* is an intracellular pathogen that establishes chronic infections through immune evasion. Exosomes, a subtype of extracellular vesicles, mediate intercellular communication and can modulate host immune responses during infection. However, the proteomic composition and functional significance of exosomes from *B. abortus*-infected macrophages remain unclear.

**Methods:**

Exosomes were isolated from RAW 264.7 macrophages infected or uninfected with *B. abortus* strain 2308, at 8 and 24 hours post-infection (hpi), using sequential centrifugation and immunoaffinity capture. Size and morphology were assessed by nanoparticle tracking analysis and transmission electron microscopy. Proteins were identified and quantified by label-free LC-MS/MS, followed by bioinformatic analyses for differential expression, functional enrichment, exclusive protein identification, and bacterial protein detection.

**Results:**

Exosomes from *B. abortus*-infected macrophages displayed distinct, time-dependent proteomic profiles. At 8 hpi, proteins involved in biosynthesis, energy metabolism, and endoplasmic reticulum processing were enriched, while lysosomal and antigen presentation components were reduced. At 24 hpi, enrichment shifted toward mitochondrial and redox regulation pathways, with sustained suppression of immune-related processes. Immune mediators (Csf3, Gsdmd, Ifi35) and retromer complex components were identified in a phase-specific manner. Sixty-six and twenty-four proteins were exclusive to infected exosomes at 8 and 24 hpi, respectively, reflecting a shift from metabolic/trafficking roles to immune regulation. Bacterial proteins GroEL and SodC were present at both time points, whereas Omp19, Omp2b, DnaK, and BAB1_0368 were restricted to early infection.

**Conclusion:**

Exosomes from *B. abortus*-infected macrophages exhibit dynamic proteomic remodeling that affects immune-related pathways, changes that may contribute to bacterial survival within the host. The presence of both host and bacterial-derived proteins within these vesicles suggests their potential relevance in brucellosis pathogenesis and highlights them as candidates worthy of further exploration as biomarkers or therapeutic targets.

## 1 Introduction

Brucellosis is a globally distributed zoonosis with a high prevalence in endemic regions of Latin America, the Middle East, Africa, and Asia (1). This disease is caused by bacteria of the genus *Brucella*, which are Gram-negative, aerobic, non-motile, and facultatively intracellular coccobacilli (2). These bacteria infect various mammalian species (3) and can be transmitted to humans through direct contact with waste derived from infected animals or through the consumption of contaminated animal products, causing chronic disease (4–6). Within the genus, *Brucella abortus* is the main etiological agent of brucellosis in cattle, representing a significant threat to animal health and, due to its proximity to humans, to global public health (7, 8). In humans, brucellosis produces a variable clinical picture, which may include undulant fever with night sweats, chronic fatigue, arthralgia, and involvement of organs such as the liver, spleen, or central nervous system (9, 10). This disease is relevant due to its symptoms and the sequelae it can cause in infected humans; but also due to the significant economic losses it causes in the agricultural industry due to abortions, infertility, and the slaughter of seropositive cattle (11–13). Brucellosis is therefore a re-emerging disease whose clinical and epidemiological complexity requires a better understanding of its mechanisms of persistence and immune evasion.

These bacteria are capable of establishing chronic infections due to their ability to survive inside phagocytic cells (macrophages and neutrophils) and to evade the host’s immune response (14). Multiple strategies have been developed by *B. abortus* to block or interrupt critical points of the innate and adaptive immune response such as phagolysosome maturation, reactive oxygen species (ROS) production, inflammatory signals, and modulate pathways involved in antigen presentation (15–18). This immunoregulatory capacity is associated with the presence of various bacterial components such as its lipopolysaccharide (Br-LPS), which presents low immunogenicity, inducing a reduced activation of TLR4 and, therefore, a lower inflammatory response (19, 20). Additionally, this bacterium has a two-component system, BvrS/BvrR, which regulates its virulence in response to changes in microenvironmental conditions such as pH or oxidative stress (21). This two-component system also controls the expression of the type IV secretion system (T4SS) VirB that translocates multiple effectors to the cytoplasm of infected cells, altering their physiology and vesicular trafficking (22). These mechanisms promote the development of a replicative niche associated with the endoplasmic reticulum, allowing this bacterium to interfere with key cellular processes that modulate the host’s immune response. Given the impact of these strategies on cellular physiology, the study of intercellular communication mechanisms, such as those mediated by extracellular vesicles, becomes especially relevant.

Exosomes are a subpopulation of extracellular vesicles (EVs) that play an essential role in intercellular communication under both physiological and pathological conditions (23). These nano-vesicles, between 30 and 150 nm in diameter, originate within multivesicular bodies (MVBs) as intraluminal vesicles (ILVs), which are released into the extracellular space after fusion of the MVB with the plasma membrane (24–26). Exosomes are composed of a lipid membrane rich in cholesterol, sphingolipids and phosphatidylserine, which encapsulates various biomolecules, including proteins, metabolites, lipids and nucleic acids, such as mRNA, microRNAs, lncRNAs and DNA (27–29). This molecular cargo reflects the physiological or pathological state of the cell of origin and can modulate processes such as cell differentiation, inflammation, angiogenesis, or the immune response in neighboring cells (30–32).

Macrophage-derived exosomes have attracted particular interest in the context of infectious diseases and immunomodulation. During bacterial infections, such as those caused by *Mycobacterium tuberculosis*, *Salmonella Typhimurium*, or *Brucella melitensis*, macrophages secrete exosomes containing pathogen-associated molecular patterns (PAMPs) such as membrane proteins, RNA, or even virulence factors (33–36). It has also been observed that in *Listeria monocytogenes* infection, exosomes are loaded with bacterial DNA capable of activating signaling mediated by the cGAS-STING cytosolic DNA sensing pathway, activating the innate immune system response (37). In the case of *Brucella*, recent studies have begun to reveal the potential of exosomes as immune modulators. Yi et al. (2021) reported that the IFITM3 protein, present in exosomes derived from infected macrophages, promotes endosomal acidification, interfering with the intracellular replication of *Brucella* (38). Complementarily, Wang et al. (2023) showed that exosomes from macrophages infected with *B. melitensis* induce polarization towards the M1 phenotype, elevating the expression of TNF-α and IFN-γ and decreasing the bacterial load in murine models (36). Thus, exosomes not only act as immune signaling vehicles, but also as antigen presentation platforms, and could be potential tools for diagnostics or development of therapeutic strategies against intracellular infections (39, 40).

Despite these advances, knowledge about the molecular content of exosomes released during *B. abortus* infection and their role in immune modulation remains limited. In this context, characterizing the content of exosomes derived from macrophages infected with *B. abortus* could provide essential clues to understanding the mechanisms of persistence and immune evasion of this bacterium. In this study, a proteomic analysis of exosomes derived from RAW 264.7 macrophages infected with *B. abortus* strain 2308 at 8 and 24 h post-infection was performed using LC-MS/MS mass spectrometry. The objective of this work was to identify host and pathogen proteins present in these vesicles to explore their potential involvement in immune modulation processes. The findings may help us better understand the strategies used by *Brucella* for intracellular persistence and could suggest new avenues for exploring the use of exosomes as biomarkers or therapeutic targets in brucellosis.

## 2 Materials and methods

### 2.1 Macrophage cell line and bacterial culture

The murine macrophage cell line RAW 264.7 (ATCC^®^ TIB71™) was cultured in Dulbecco’s Modified Eagle’s Medium (DMEM) (Life Technologies^®^), supplemented with 10% exosome-depleted serum (FBS) (GibcoTM, USA) and 1% antibiotic-antimycotic solution (Mediatech, Inc.), and incubated at 37°C and 5% CO_2_. On the other hand, bacterial cultures of the virulent strain *B. abortus* 2308, provided by the strain library of the Molecular Immunology Laboratory at the University of Concepción, were grown on Brucella agar (BD, USA) at 37°C for 72 h. Liquid cultures were generated from the isolated colonies by seeding onto Brucella broth (BD, USA), which were then incubated at 37°C for 72 h under shaking. All work with live bacteria was conducted in biosafety level 2 facilities, following all guidelines of the Institutional Bioethics and Biosafety Committee of the University of Concepcion (Certificate CEBB 1466-2023).

### 2.2 Macrophage infection assays with *B. abortus* 2308

RAW 264.7 macrophages were maintained in 75 cm^2^ cell culture flasks (SPL, Gyeonggi-do, Korea) under culture conditions. Once the desired confluence was reached, cells were harvested, washed three times with PBS, and their number and viability were determined using a Countess Automated Cell Counter 3 (Thermo Fisher Scientific Inc., Waltham, MA, USA) using the Trypan Blue exclusion assay. Subsequently, approximately 1 x 10^8^ cells suspended in 200 ml of DMEM medium were seeded per 5-tier cell culture flask (870 cm2) (NEST, Jiangsu, China) (41), incubating for 2 h to ensure cell adherence to the plates. Once the RAW 264.7 macrophages adhered, they were infected with *B. abortus* 2308 at a multiplicity of infection (MOI) of 1:100 (42), incubating for 1 h at 37°C in a 5% CO_2_ atmosphere. In order to remove extracellular bacteria, the cells were washed 3 times, with gentle agitation with PBS supplemented with 100 μg/ml of gentamicin for 5 min and subsequently 3 times with PBS alone. Finally, the PBS was replaced with DMEM supplemented with 100 μg/ml gentamicin, and the cells were incubated for an additional 8 or 24 h. For the uninfected condition, the medium was removed, and the cells were incubated for 1 h with DMEM, washed with PBS, and incubated again in DMEM for an additional 8 or 24 h. Three independent replicates were performed for each condition.

### 2.3 Exosome purification

The medium derived from the culture of RAW 264.7 macrophages under control and infected conditions from each replicate was collected individually and then subjected to sequential centrifugation to remove cell debris: for 5 min at 300 × g, 20 min at 1,200 × g, and finally for 30 min at 10,000 × g, at 4°C. The supernatant was then filtered through a 0.22 µm pore size (Cytiva, Uppsala, Sweden), concentrated in Vivaspin^®^ 20 ultrafiltration units (Cytiva, Uppsala, Sweden) and centrifuged at 6,500 × g for 40 min at 4°C, ultimately yielding a volume of approximately 500 µL. Exosome recovery from this concentrated material was achieved using the MagCapture™ Exosome Isolation Kit PS Ver. 2 (FUJIFILM Wako Pure Chemical Corporation, USA) following manufacturer instructions. Briefly, Biotin Capture Magnetic Beads were prepared by washing with 500 µL of Exosome Capture Immobilizing/Washing Buffer. Biotin-labeled Exosome Capture Reagent was then added and incubated for 10 min at 4°C. The concentrated sample was diluted in Exosome Binding Enhancer 500X, transferred to tubes containing magnetic beads, and incubated for 12 h at 4°C with constant agitation. Subsequently, the supernatant was recovered, and exosomes were eluted using 50 µL of Exosome Elution Buffer 1X. The recovered medium was reincubated with the magnetic beads in three additional cycles, with incubation times of 2, 1, and 1 h, respectively. Finally, all elutions were collected and pooled into a final fraction.

### 2.4 Exosome characterization

Purified exosomes were characterized using parameters such as morphology and size (29). To determine the average size and particle concentration, nanoparticle tracking analysis (NTA) was performed using the NanoSight NS300 instrument (Malvern Panalytical, USA). Samples were diluted 1:30 with sterile PBS, injecting 50 µL into the instrument. Particle tracking was set within a range of 10–100 particles per frame. Data were captured and analyzed using NTA analytical software (version 3.2, Dev Build 3.2.16). The morphology and structure of the vesicles were evaluated by transmission electron microscopy (TEM). For this purpose, 5 µL of the exosome sample were deposited on a copper grid coated with formvar-carbon, incubated for 5 min, and subsequently stained with 2% phosphotungstic acid (pH 7.4). Images were obtained using a JEOL JEM-1200 EX electron microscope operating at 80 kV.

### 2.5 Protein extraction for LC-MS/MS

The exosome samples were lyophilized and resuspended in 500 µL of a solution of 8 M urea and 25 mM ammonium bicarbonate. These samples were then disrupted by sonication for 2 min, applying 10-s pulses at 40% intensity. The samples were subsequently alkylated by adding 20 mM iodoacetamide dissolved in 25 mM ammonium bicarbonate, incubated in the dark for 30 min at room temperature, and then precipitating the proteins with a methanol/chloroform solution. To do this, one volume of the extract was mixed with five volumes of 100% methanol, followed by the addition of one volume of 99% (v/v) chloroform. The resulting mixture was homogenized by gentle shaking, followed by addition the three volumes of Milli-Q water. The mixture was centrifuged at 15,000 × g for 5 min, allowing the formation of a visible intermediate phase corresponding to the concentrated proteins. Finally, the extracts were washed four times with 400 µL of 100% methanol and dried in a rotary concentrator at 2,000 rpm overnight at 40°C. To prepare the samples for LC-MS/MS analysis, enzymatic digestion was performed using sequencing-grade trypsin (Promega, cat. no. V5071) at a 1:50 ratio (protease/protein, mass/mass) for 16 h at 37°C. The digestion reaction was stopped by acidification with 10% (v/v) formic acid, reaching a pH of approximately 2.0. Finally, 200 ng of peptides were purified using disposable C18 Evotips columns (EVOSEP Biosystems). The purified peptides were analyzed by liquid chromatography coupled with tandem mass spectrometry (LC-MS/MS) using an Evosep One system (Evosep Biosystems) coupled to a timsTOF Pro 2 mass spectrometer (Trapped Ion Mobility Spectrometry – Quadrupole Time-of-Flight, Bruker Daltonics). The system employed an EVOSEP Performance column (15 cm × 150 µm, 1.5 µm ReproSil-Pur C18, Evosep Biosystems) for chromatographic separation. The analysis was performed using the 30 SPD (samples per day) method, applying an elution gradient of 2% to 35% buffer B (acetonitrile with 0.1% formic acid). Data acquisition was done using TimsControl 2.0 software (Bruker Daltonics), operating in PASEF (Parallel Accumulation–Serial Fragmentation) mode with 10 cycles per acquisition, in a mass range of 100–1,700 m/z. Electrospray ionization was performed at a voltage of 1,500 V, with a capillary temperature of 180°C. The spectrometer operated at a TOF frequency of 10 kHz, with a resolution of approximately 50,000 FWHM (Full Width at Half Maximum) (43).

### 2.6 Protein identification

For protein identification, the obtained spectral data were analyzed using MSFragger v4.1 software (44) run through the FragPipe v22.0 platform (https://fragpipe.nesvilab.org), using the default workflow. A high-performance server with 48 processing cores and 512 GB of RAM was used for the analysis. The established parameters included a mass tolerance for precursors of –20 to +20 ppm and a mass tolerance for fragments of 40 ppm. For in silico digestion, trypsin was used as the enzyme, under specific digestion mode, allowing for up to two missed cleavages per peptide. Post-translational modifications (PTMs) were defined as fixed modifications: carbamidomethylating of cysteine residues; and variable modifications: methionine oxidation (M) and acetylation at the N-terminus. The search was performed against a combined database composed of the *Mus musculus* proteome (UniProt code: UP000005640) and the *Brucella abortus* proteome (UniProt code: UP000002719). A database of common contaminants in mass spectrometry was included to improve the quality of the filtering. Statistical control of the analysis was performed by applying an FDR (False Discovery Rate) estimate of <1%, using a decoy database for identification validation (44).

### 2.7 Comparison of protein profiles

Proteins identified in exosomes derived from uninfected (EXOC) and infected (EXOI) macrophages were compared at two incubation times (8 or 24 h post-infection (hpi)). For this purpose, proteins were considered exclusive proteins if they were detected only in one of the two experimental conditions (EXOC or EXOI) in the three assays performed (biological replicates). The sets of shared and exclusive proteins were visualized using Venn diagrams, generated with the Venn Diagram Tool web platform of the VIB Bioinformatics Core (http://bioinformatics.psb.ugent.be/webtools/venn).

### 2.8 Protein subcellular localization analysis

The sequenced protein IDs were converted to UniProt IDs (45) and subsequently analyzed using the UniProt Batch Retrieval Tool (https://www.uniprot.org/) to obtain annotated subcellular localization. The data were processed and categorized according to major cellular compartments: cytoplasm, nucleus, plasma membrane, cytoskeleton, mitochondria, endoplasmic reticulum (ER), Golgi apparatus, and intracellular membranes other than mitochondria and Golgi. This last category includes proteins located in endosomes, transport vesicles, and endomembrane structures not classified in the previous categories. The results were expressed as a percentage of the total proteins with defined localization. The graphical representations were generated using the SRPlot platform (https://www.bioinformatics.com.cn).

### 2.9 Protein quantification by label-free quantification

For relative protein quantification, the intensity values from each run were used, which were normalized by adjusting the medians, equalizing them to a common value to reduce systematic variability between samples (46). Missing values were handled using the MissForest algorithm, a nonparametric imputation method based on random forests that allows estimating missing values within each experimental condition (47). As an inclusion criterion, only proteins detected in at least two of three biological replicates corresponding to each experimental group were considered. Differentially expressed proteins between conditions were identified by applying a linear model together with a Bayesian moderated t-test, using the R package limma (48). Proteins with a p-value < 0.05 were considered significant. The primary comparison in this study was between proteins in the infected versus uninfected conditions, considering both post-infection times. Graphical representations related to the quantification of the results were generated in R (3.6.0), using the EnhancedVolcano (49) and Complex Heatmap v.2.0.0 (50) packages.

### 2.10 Functional enrichment analysis (Gene Ontology and KEGG)

To explore the biological processes associated with the proteins of interest, a functional enrichment analysis was performed using the Gene Ontology (GO) database and the KEGG (Kyoto Encyclopedia of Genes and Genomes) pathway database. Both analyses were conducted using the Enrichr web platform (51). The results included significantly enriched terms for biological functions, cellular components, molecular processes, and metabolic pathways, with a *P*-value < 0.05 considered significant. Graphical representations of the enriched terms were generated using the SRPlot platform (https://www.bioinformatics.com.cn).

### 2.11 Analysis of unique proteins, interaction networks, and functional annotation

The unique proteins of exosomes derived from *B. abortus*-infected macrophages (EXOI) were determined by comparing the pan-proteome of control exosomes (EXOC) with the core-proteome of EXOI. The pan-proteome was defined as the total set of proteins identified in at least one replicate, while the core-proteome considered those present in all three biological replicates. Intersections and uniqueness were visualized using Venn diagrams generated using the VIB/UGent Bioinformatics Web Tools platform (http://bioinformatics.psb.ugent.be/webtools/venn). EXOI-unique proteins at 8 and 24 hpi were analyzed using functional interaction networks using the STRING database (https://string-db.org/), selecting *Mus musculus* as the reference organism and applying a medium confidence threshold (score > 0.4). Interactions based on experimental and database evidence, co-expression, and co-occurrence were considered. Additionally, functional annotation was performed using enriched terms from Reactome pathway database and the Gene Ontology (GO), specifically in the Biological Process (BP) category. The results were organized into chord diagrams showing the relationship between unique proteins and their associated biological functions. The visualizations were generated using the RAWGraphs platform (https://www.rawgraphs.io), using the chord diagram to highlight multiple functional connections.

### 2.12 Identification of *B. abortus* proteins in exosomes

To confirm the presence of bacterial proteins in EXOC and EXOI, proteins annotated with *Brucella abortus* 2308 (UNIPROT ID: UP000002719) were identified. After identifying these proteins, their subcellular localization was predicted using the PSORTb v3.0 server (https://psort.org/psortb/), a tool specifically for prokaryotic proteomes. PSORTb predicts bacterial subcellular localization (cytoplasm, inner membrane, periplasm, outer membrane, or extracellular membrane) using a rule-based system and machine learning trained with experimental proteomic data (52).

**Figure 1:**
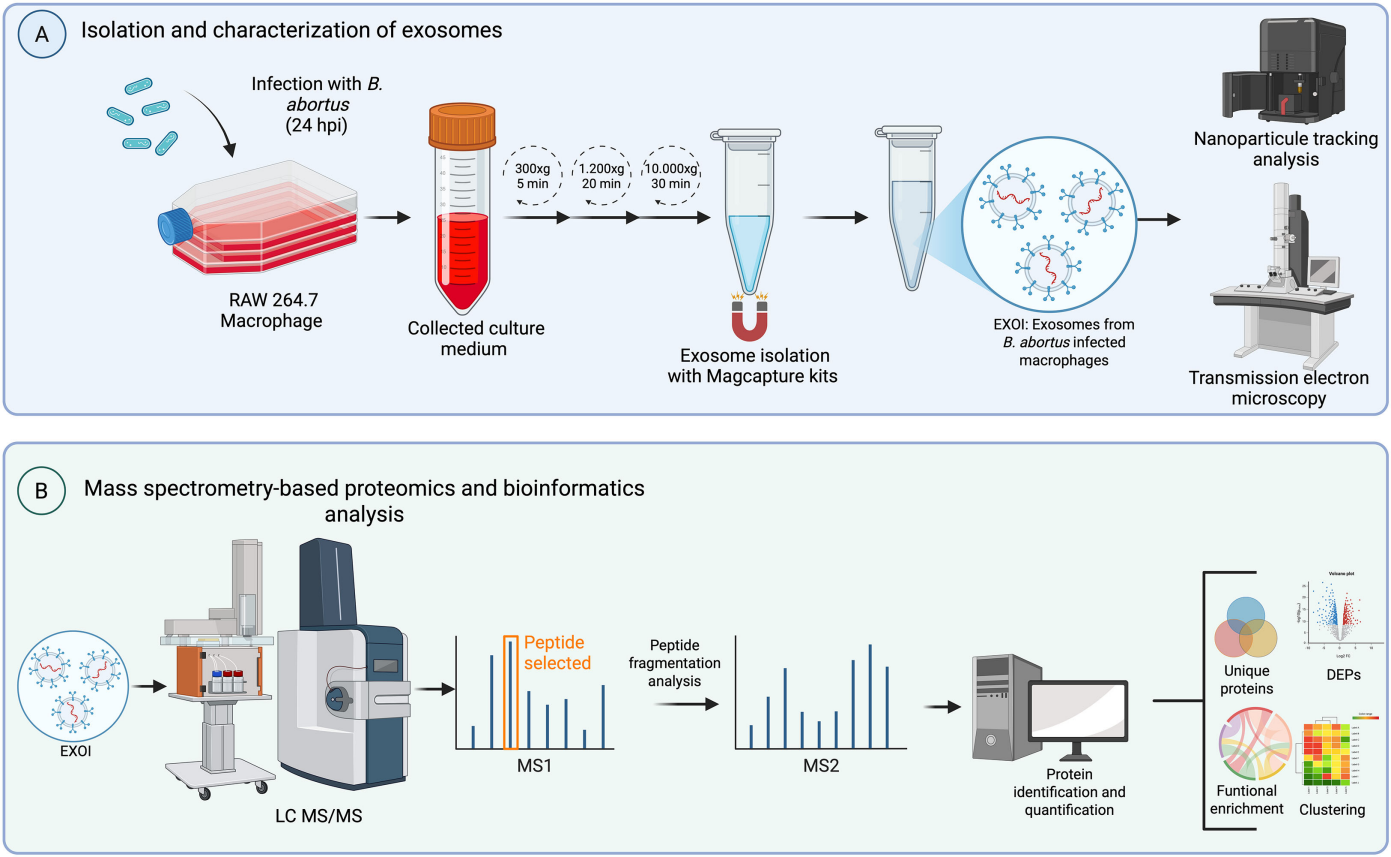
Experimental workflow. Schematic overview of the experimental design used to analyze proteomic composition for exosomes derived from *Brucella abortus*-infected macrophages. **(A)** RAW 264.7 macrophages were infected with *B*. *abortus* and culture supernatants were collected at two time points (8 or 24 hpi). Exosomes were isolated from the conditioned medium through differential centrifugation and MagCapture™ Exosome Isolation Kit PS VER. 2 (FUJIFILM Wako Pure Chemical Corporation, USA). Purified exosomes were characterized by nanoparticle tracking analysis (NTA) and transmission electron microscopy (TEM). **(B)** Exosomal proteins were subjected to liquid chromatography-tandem mass spectrometry (LC-MS/MS). The resulting data were processed for peptide identification, quantification, and downstream bioinformatic analyses including differential expression (DEPs), detection of unique proteins, clustering, and functional enrichment.

The methodological pipeline described in the previous sections is graphically summarized in **Figure 1**, which provides an overview of the experimental design and analytical steps.

## 3 Results

### 3.1 Characterization of RAW 264.7 macrophage-derived exosomes

**Figure 2:**
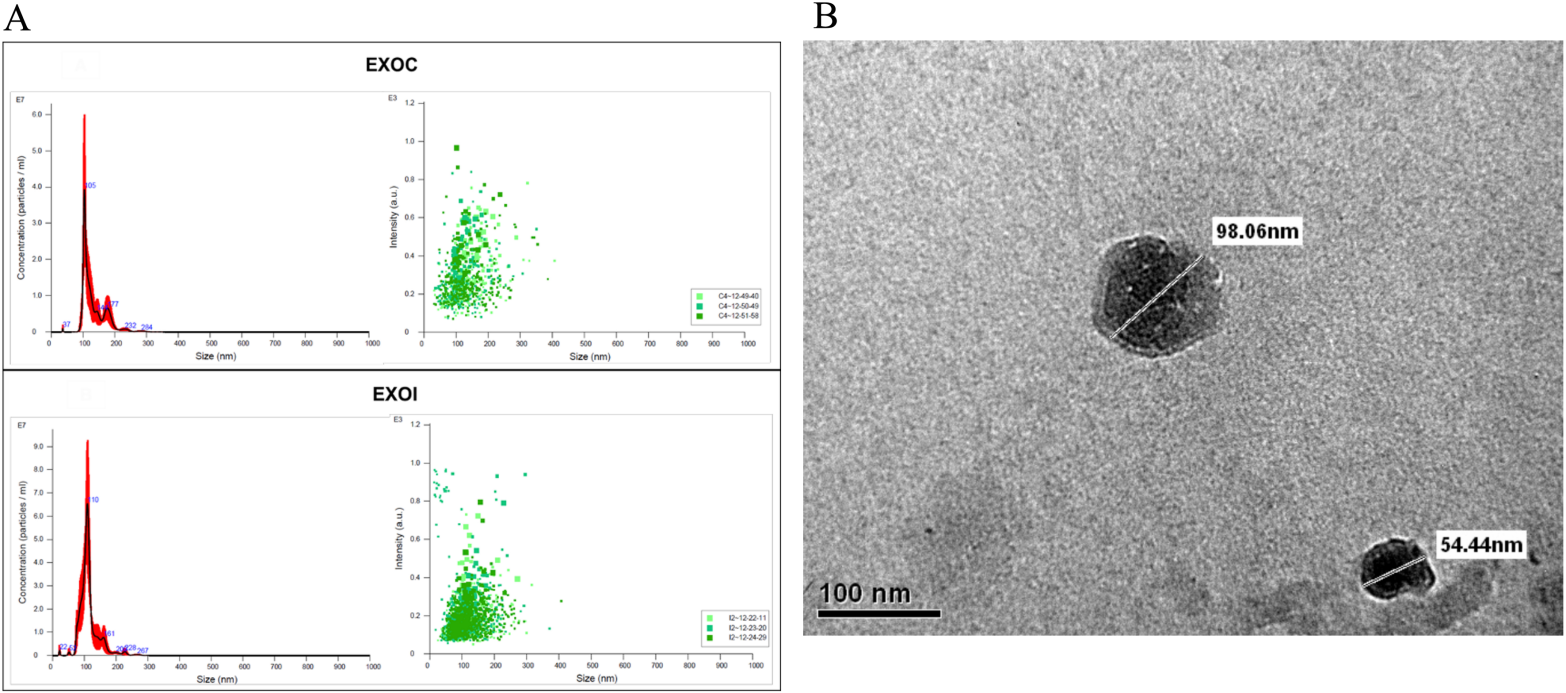
Characterization of exosomes derived from RAW 264.7 macrophages. **(A)** Nanoparticle tracking analysis (NTA) of exosomes obtained from control (EXOC) and *B*. *abortus* 2308-infected (EXOI) macrophages, showing particle size distribution, concentration (left), and intensity (right); **(B)** Transmission electron microscopy (TEM) of purified vesicles derived from *B*. *abortus* 2308-infected macrophages.

Extracellular vesicles were characterized according to criteria of size and morphology. Nanoparticle tracking analysis (NTA) showed comparable size profiles between vesicles from the control group (EXOC), with a size distribution peak at 105 nm, and those from *the B. abortus* 2308-infected group (EXOI), which peaked at 110 nm (**Figure 2A**). Extracellular vesicles was analyzed by transmission electron microscopy (TEM), revealing circular structures with a size between 54 and 98 nm, characteristic of exosomes (**Figure 2B**). By analyzing the size and structural integrity of the vesicles, we confirmed that we were isolating exosomes, and then determined their proteomic composition under infected and uninfected conditions.

### 3.2 Exosome proteome identification

**Figure 3:**
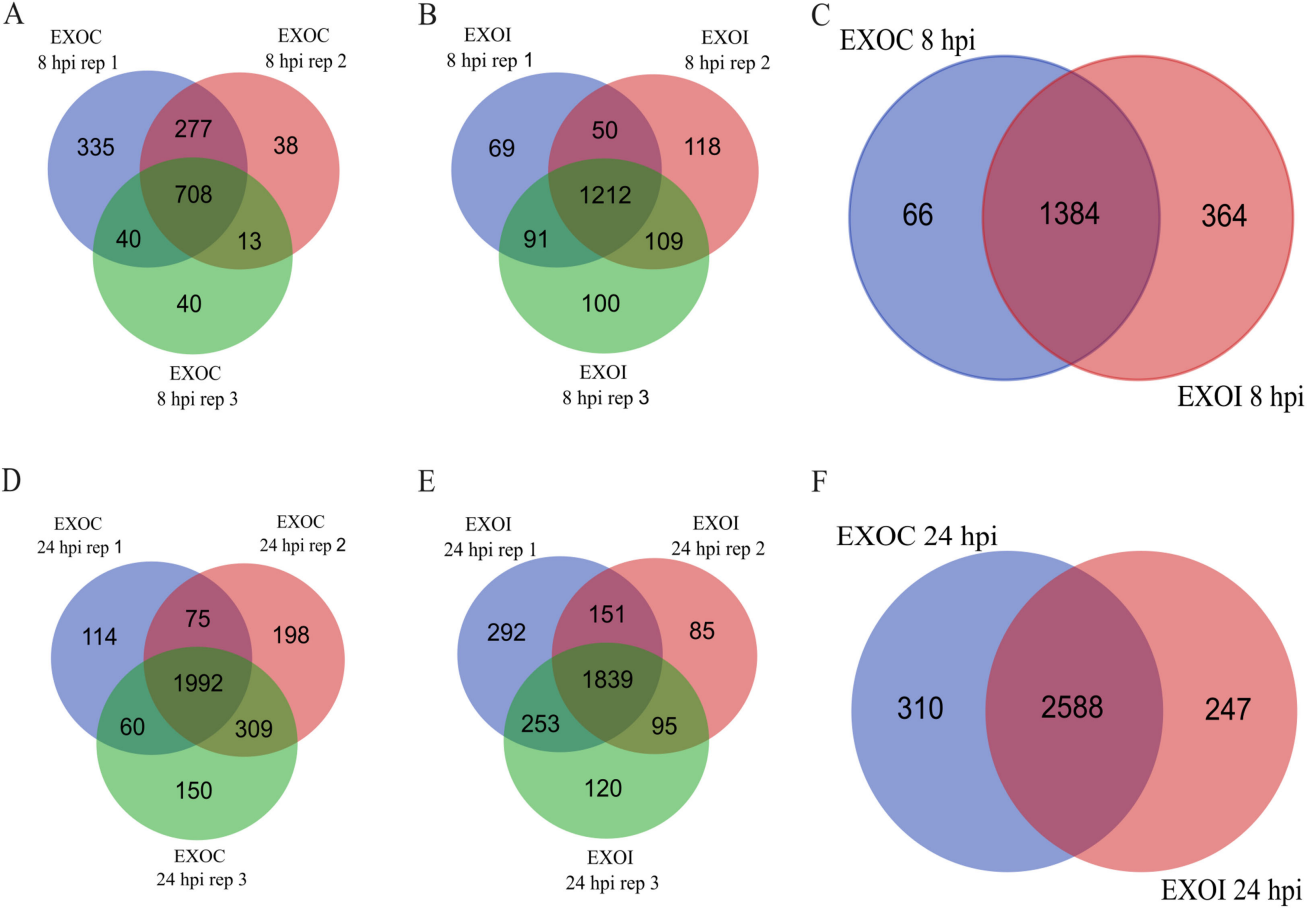
Comparison of proteins identified in exosomes from control macrophages (EXOC) and those infected with *B*. *abortus* 2308 (EXOI). Venn diagrams show the number of proteins identified in each biological replicate for EXOC **(A)** and EXOI **(B)** at 8 hpi. **(C)** Comparison of the total set of identified proteins between both conditions (EXOC vs. EXOI). Venn diagrams corresponding to 24 hpi show the distribution of proteins between replicates for EXOC **(D)**, EXOI **(E)** and the comparison between both conditions **(F)**.

The proteome of exosomes derived from uninfected RAW 264.7 macrophages (EXOC) and exosomes released by infected macrophages (EXOI) was analyzed using liquid chromatography-mass spectrometry (LC-MS/MS). At 8 hpi, 1,450 proteins were identified in EXOC, with 708 (48.8%) shared across the three biological replicates (**Figure 3A**). A total of 1,748 proteins were identified in EXOI, of which 1,212 (69.3%) were detected in all three replicates (**Figure 3B**). Both control and infected conditions were compared, with 1,384 common proteins were detected for both conditions, while 66 proteins were unique to EXOC (3.63%) and 364 unique to EXOI (20.1%) out of a combined total of 1,814 proteins (**Figure 3C**). At 24 hpi, 2,898 proteins were identified in EXOC and 2,835 in EXOI, with 68.7% and 64.9% agreement between replicates, respectively (**Figures 3D** and **2E**). In the comparison between conditions, 2,588 shared proteins were identified, 310 unique to the control group and 247 unique to the infected group, representing a combined total of 3,145 proteins (**Figure 3F**). To strengthen the reliability of these comparisons, we also analyzed the core proteome, defined as proteins consistently identified across all three biological replicates. At 8 hpi, 708 proteins were reproducibly detected in EXOC and 1,212 in EXOI, whereas at 24 hpi, 1,992 proteins were consistently identified in EXOC and 2,202 in EXOI. Comparative analyses based on these core sets are shown in the supplementary Venn diagrams (**Supplementary Figures 1A, B**). To validate the exosomal nature of the analyzed vesicles, we confirmed the presence of classical exosomal markers CD9, CD63, and CD82 by proteomic analysis at both 8 hpi and 24 hpi, in exosomes derived from both infected and uninfected macrophages (**Supplementary Table 1**). To further explore the origin and potential functions of the identified proteins, we examined their predicted subcellular localization.

### 3.3 Subcellular localization analysis

**Figure 4:**
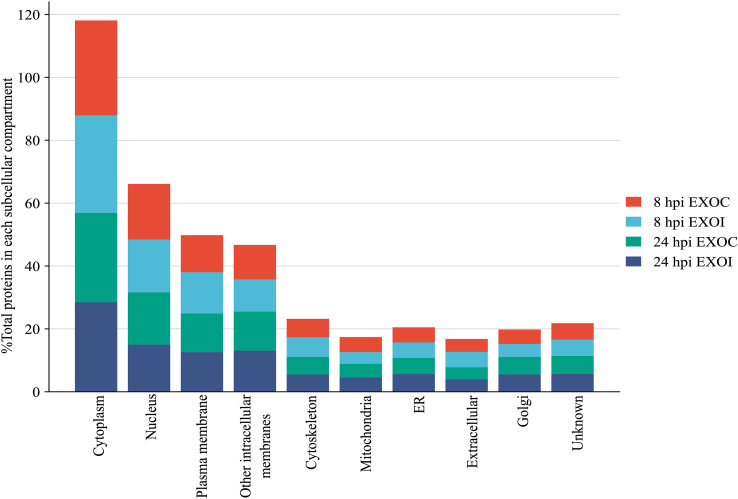
Subcellular localization analysis. Distribution of total proteins identified according to their subcellular localization, expressed as percentages of the total proteins with defined localization. Results are presented for each experimental condition: EXOC (red and green) and EXOI (light blue and blue) at the corresponding analysis times (8 hpi and 24 hpi).

A subcellular localization analysis of the proteins identified in exosomes derived from RAW 264.7 macrophages at 8 hpi showed that in both groups, the majority of the proteins identified were cytoplasmic (30.2% in EXOC and 31.0% in EXOI), followed by proteins with nuclear localization (17.7% in EXOC and 16.8% in EXOI), and plasma membrane localization (11.8% in EXOC and 13.1% in EXOI). Proteins grouped into the “other intracellular membranes” category were identified as well (11.0% in EXOC and 10.2% in EXOI), which includes proteins located in structures belonging to the endomembrane system such as endocytic vesicles and lysosomes, excluding proteins associated with the endoplasmic reticulum (ER) and Golgi apparatus, which are considered in separate categories. On the other hand, at 24 hpi a higher proportion of proteins associated with the cytoplasm was observed (28.5% in EXOC and 28.4% in EXOI), followed by proteins located in the nucleus (16.6% in EXOC and 15.0% in EXOI), in the plasma membrane (12.3% in EXOC and 12.6% in EXOI) and in other intracellular membranes (12.4% in EXOC and 13.1% in EXOI). Additionally, in both groups proteins associated with the cytoskeleton, mitochondria, endoplasmic reticulum, extracellular compartments, Golgi apparatus and a residual percentage of unannotated proteins with undefined localization were detected as well (**Figure 4**). Then, we determined how infection dynamically alters the exosomal protein content by performing differential expression analysis.

### 3.4 Differential protein expression analysis

**Figure 5:**
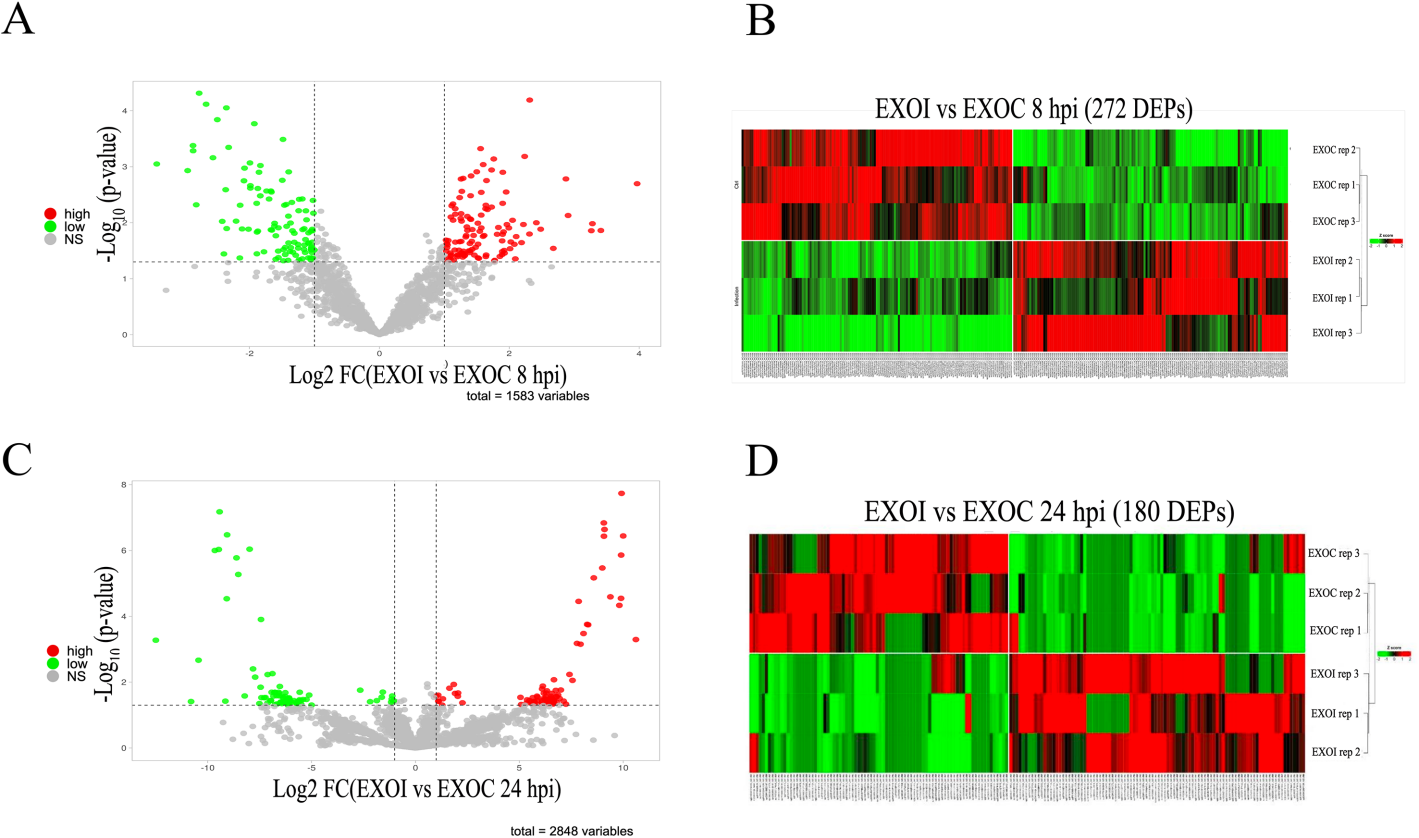
Differential protein expression analysis (DEPs) in exosomes derived from control (EXOC) and *B*. *abortus* 2308-infected (EXOI) macrophages. **(A)** Volcano plot representing differentially expressed proteins (DEPs) between EXOI and EXOC at 8 hpi. Overexpressed proteins in EXOI are shown in red (log_2_FC > 1, P < 0.05), underexpressed proteins in green (log_2_FC < –1, P < 0.05), and non-significant proteins in gray. **(B)** Heat map with hierarchical clustering analysis showing the expression patterns of the 272 DEPs identified at 8 hpi. Columns correspond to biological replicates and rows to proteins. Colors indicate relative expression levels: red (high), green (low). **(C)** Volcano plot corresponding to the analysis at 24 hpi. The DEPs between EXOI and EXOC are shown under the same statistical criteria. **(D)** Heat map with hierarchical clustering analysis of the 180 DEPs at 24 hpi. Color intensities represent relative expression levels, where red represents overexpression and green indicates underexpression.

Comparative analysis of differential protein expression between exosomes from infected (EXOI) and uninfected (EXOC) macrophages at 8 hpi identified 272 DEPs, using adjusted P < 0.05 and a fold change in expression with |Log_2_FC| > 1 as criteria (**Figure 5A**, **Supplementary Table 2**). Hierarchical clustering analysis showed a clear separation between infected and uninfected conditions (**Figure 5B**). Comparative analysis at 24 hpi identified 180 DEPs expressed between EXOI and EXOC, using the same statistical criteria described above (adjusted P < 0.05 and |Log_2_FC| > 1) (**Figure 5C**, **Supplementary Table 3**). Hierarchical analysis distinguished both experimental conditions based on their expression profiles (**Figure 5D**). To better interpret the differential protein expression results, we conducted functional enrichment analysis of the DEPs using GO and KEGG databases.

### 3.5 Functional analysis of differentially expressed proteins in exosomes

**Figure 6:**
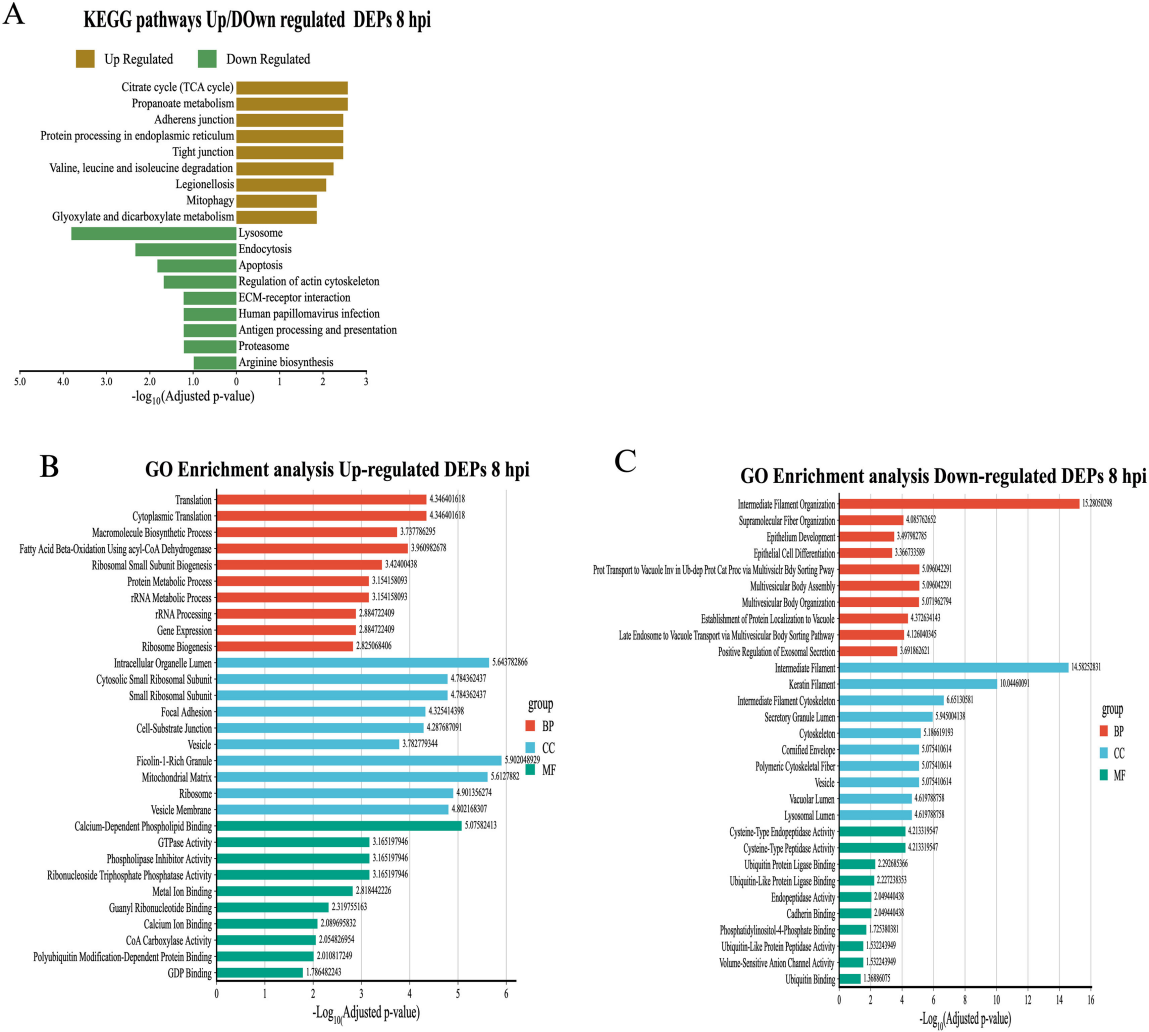
Functional enrichment analysis of differentially expressed proteins (DEPs) in exosomes derived from RAW 264.7 macrophages infected with *B*. *abortus* at 8 hpi. **(A)** KEGG functional pathway enrichment for overexpressed (brown bars) and underexpressed (green bars) proteins at 8 hpi. **(B)** Gene Ontology (GO) term enrichment analysis for overexpressed proteins at 8 hpi, under the categories of Biological Process (BP), Cellular Component (CC), and Molecular Function (MF). **(C)** GO term enrichment for underexpressed proteins at 8 hpi, grouped by ontology category.

To understand the biological functions associated with differentially expressed proteins (DEPs) in exosomes derived from macrophages infected with *B. abortus*, a functional enrichment analysis was performed using the KEGG and Gene Ontology (GO) databases. The results showed dynamic alterations in pathways linked to cellular metabolism, protein processing, and immune functions throughout the infection. At 8 hpi, KEGG analysis revealed overexpression of exosomal proteins associated with protein biosynthesis (Ribosome), energy metabolism (TCA cycle, Propanoate metabolism), and protein processing in the endoplasmic reticulum (Protein processing in endoplasmic reticulum). Pathways related to cell junction remodeling and mitochondrial recycling (Mitophagy) were also identified (**Figure 6A**). These findings were consistent with the GO analysis, in which the enriched biological processes corresponded mainly to “Translation” and “Macromolecule biosynthetic process.” In the cellular component category, a high representation of ribosomal, mitochondrial, and vesicular structures was observed. Overexpressed molecular functions included terms related to GTPase activity, signaling, vesicular dynamics, and ubiquitin-like protein binding (**Figure 6B**). In contrast, analysis of underexpressed proteins revealed a negative modulation of key immune pathways, such as “Lysosome,” “Endocytosis,” “Antigen processing and presentation,” and “Proteasome” (**Figure 6A**). GO enrichment reinforced these results, highlighting terms such as “Intermediate filament organization,” “Multivesicular body organization,” and “Positive regulation of exosomal secretion.” The underexpressed proteins were associated with cellular components such as the lysosomal lumen, cytoskeleton, and secretory vesicles, and showed a decrease in molecular functions associated with endopeptidase activities and binding to ubiquitin-like proteins (**Figure 6C**). At 24 hpi, a reconfiguration of the functional profile was evident.

**Figure 7:**
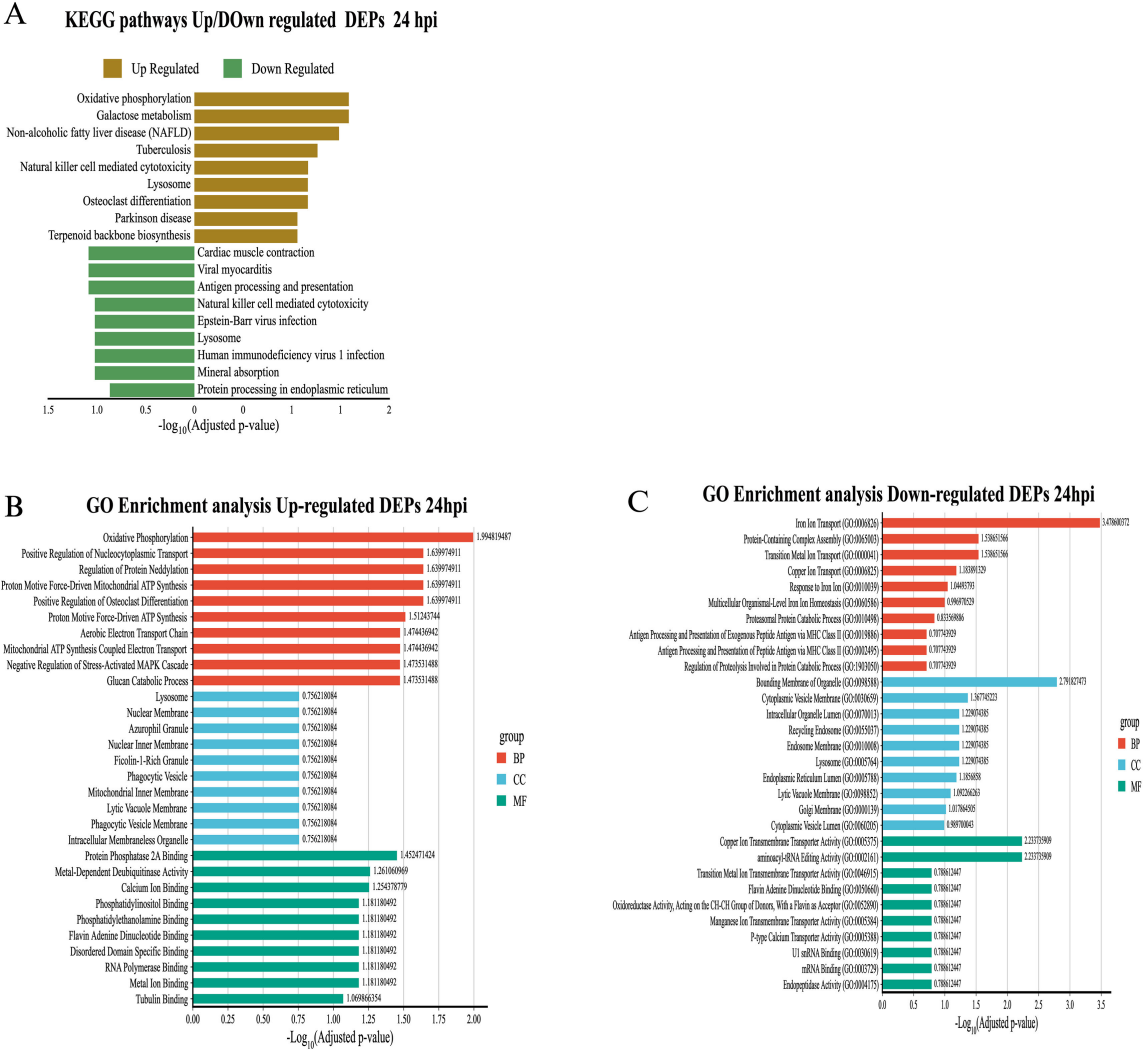
Functional enrichment analysis of differentially expressed proteins (DEPs) in exosomes derived from RAW 264.7 macrophages infected with *B*. *abortus* at 24 hpi. **(A)** KEGG pathway enrichment for differentially expressed proteins at 24 hpi. **(B)** GO enrichment for overexpressed proteins at 24 hpi. **(C)** GO enrichment for underexpressed proteins at 24 hpi. Significance values are presented as –log_10_ of the adjusted p-value, and colors indicate GO category: red for biological processes (BP), blue for cellular components (CC), and green for molecular functions (MF).

KEGG analysis showed an enrichment of mitochondrial metabolic pathways (“Oxidative phosphorylation”, “Galactose metabolism”) and cellular differentiation (“Osteoclast differentiation”), as well as processes related to metabolic diseases (“Non-alcoholic fatty liver disease”) (**Figure 7A**). These results were supported by GO analysis, where terms such as “Oxidative phosphorylation”, “Proton motive force-driven ATP synthesis”, and “Aerobic electron transport chain”, all related to ATP synthesis in mitochondria, stood out. From an immunological perspective, GO analysis showed an increase in proteins located in key structures such as lysosome, phagocytic vesicle, and ficolin-1-rich granule. Enriched molecular functions such as metal ion binding, protein phosphatase 2A binding, and calcium ion binding, associated with cell signaling processes, were observed as well (**Figure 7B**). On the other hand, underexpressed proteins at 24 hpi showed a decrease in essential pathways for immune response and host defense. In KEGG, significant reductions were observed in “Proteasome” and “Antigen processing and presentation,” as well as in pathways related to “Lysosome,” “Endocytosis,” and “Apoptosis” (**Figure 7A**). GO analysis complemented these findings, showing negative enrichment in metal ion transport pathways (“Iron ion transport”, “Copper ion transport”, “Transition metal ion transport”) and protein degradation processes (“Proteasomal protein catabolic process”, “Regulation of proteolysis involved in protein catabolic process”). Terms associated with antigenic processing by MHC class II also decreased. The underexpressed proteins were localized to cellular components such as “Lysosome”, “Endosome membrane”, “Cytoplasmic vesicle membrane” and “Endoplasmic reticulum lumen”. As soon as molecular functions, a reduction in ion transport activities, RNA processing and transcriptional regulation was observed (**Figure 7C**). In addition to differentially expressed proteins, we also identified proteins exclusively present in exosomes from infected macrophages, which may represent infection-specific signature.

### 3.6 Functional analysis of proteins exclusive to exosomes derived from infected macrophages

**Figure 8:**
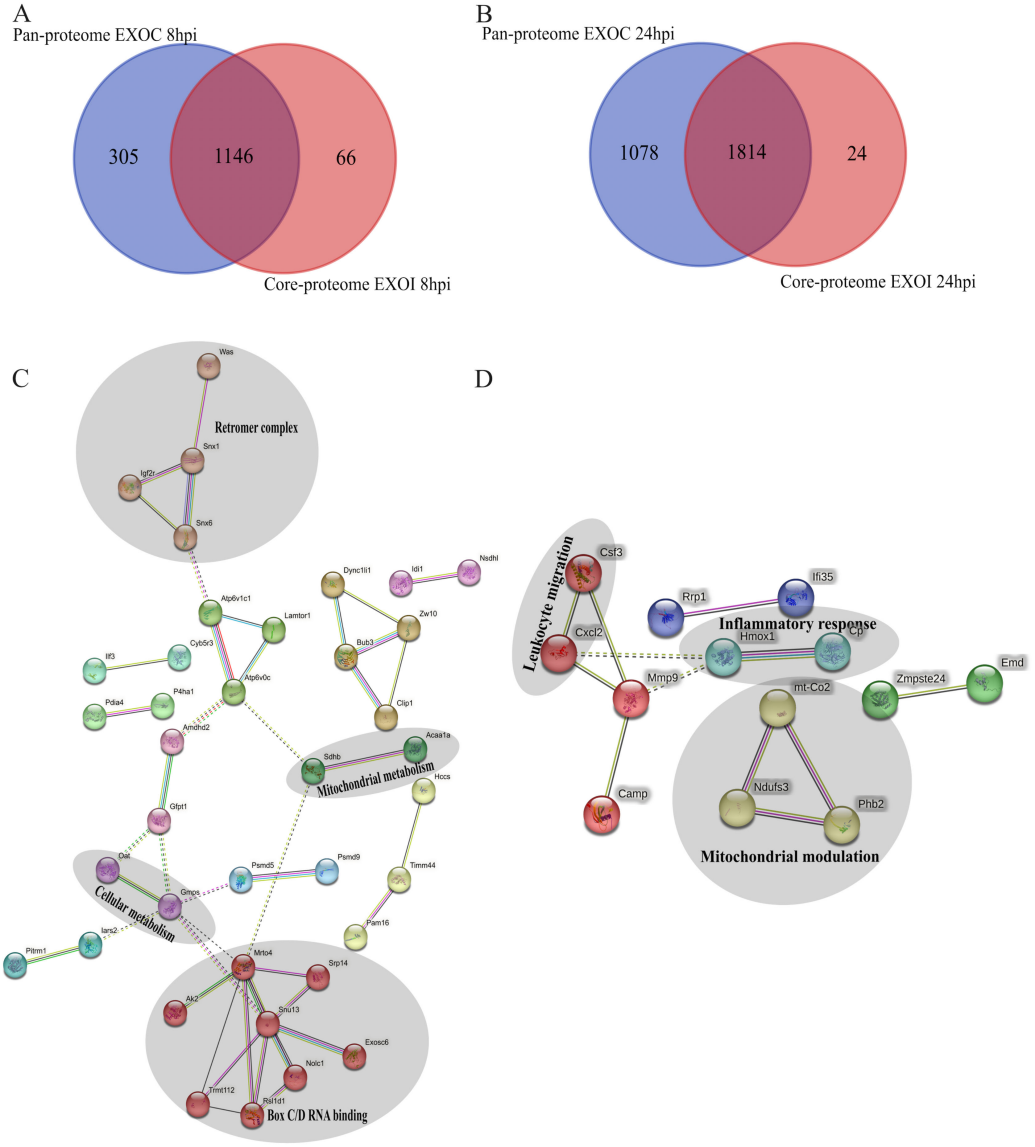
Functional analysis of unique proteins in exosomes derived from *B*. *abortus*-infected macrophages at 8 and 24 hpi. **(A, B)** Venn diagram showing the unique proteins detected in exosomes from infected macrophages (EXOI) compared to control exosomes (EXOC) at 8 hpi **(A)** and 24 hpi **(B)**. **(C, D)** Protein-protein interaction (PPI) network generated in STRING for EXOI-unique proteins at 8 hpi (n=66) and 24 hpi (n=24).

**Figure 9:**
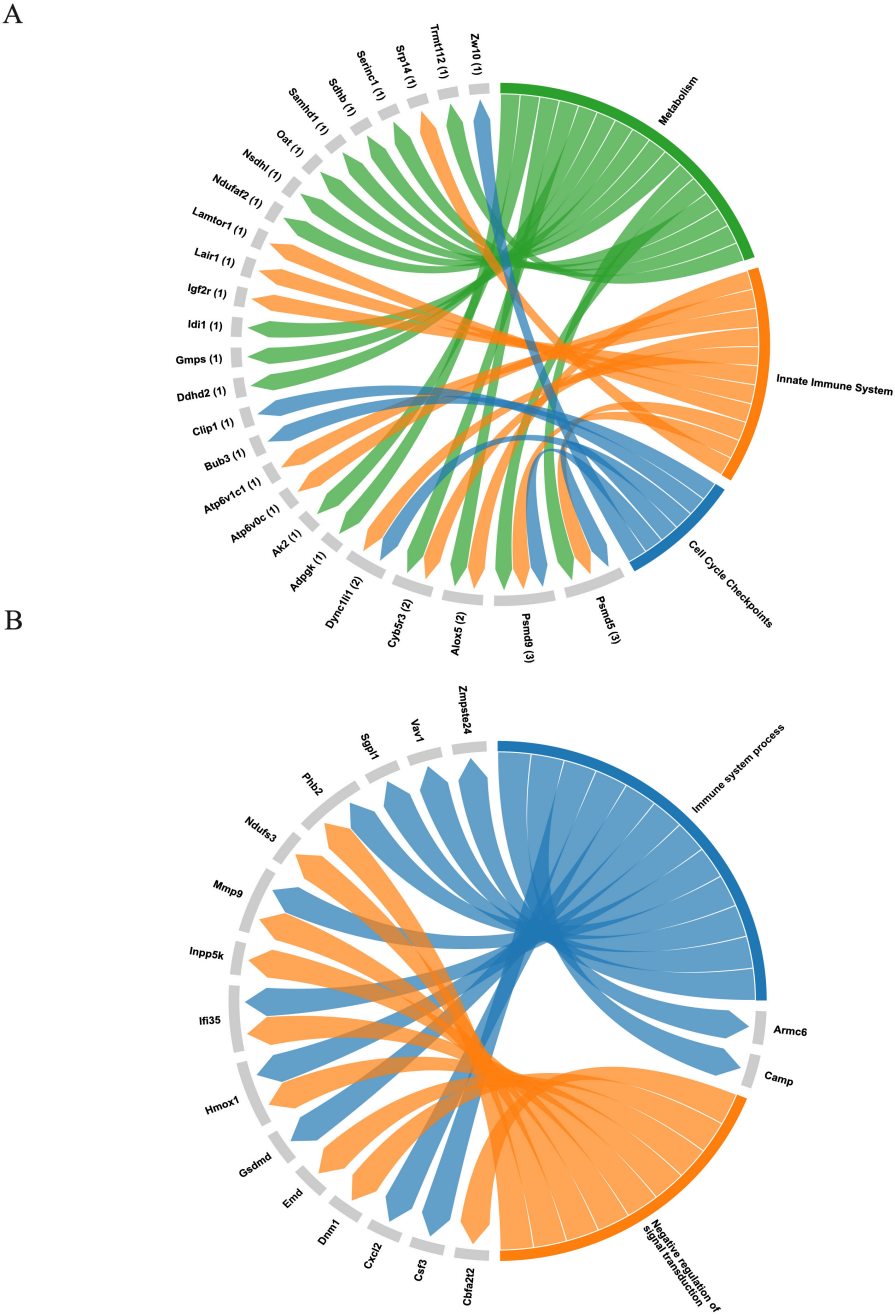
Functional representation of unique proteins in exosomes derived from *B*. *abortus*-infected macrophages. **(A)** Chord diagram showing the association between the sixty-six exclusive proteins at 8 hpi and significantly enriched pathways from the Reactome database, including metabolism, sister chromatid separation, innate immune system, and neutrophil degranulation. **(B)** Chord diagram corresponding to the twenty-four exclusive proteins at 24 hpi, based on Gene Ontology: Biological Process (GO: BP) terms, highlighting processes related to immune response and negative regulation of signal transduction.

A total of sixty-six proteins were identified exclusively in exosomes derived from *B. abortus*-infected macrophages at 8 hpi, and twenty-four exclusive proteins were detected at 24 hpi (**Figures 8A, B**, respectively). These proteins were consistently found in all three replicates of exosomes derived from *B. abortus*-infected macrophages (EXOI) and were absent in all replicates of control exosomes (EXOC). Functional interaction network analysis (STRING) revealed marked differences between both time points. At 8 hpi (**Figure 8C**), the exclusive proteins were organized into multiple functional clusters. The main cluster (red) included proteins of the Box C/D ribonucleoprotein complex (Snu13, Mrto4, Rsld1, Trmt112), involved in rRNA processing and ribosomal assembly. Another cluster (brown) corresponded to the retromer complex, including Snx1, Snx6, and Igf2r. Additional modules were related to cellular metabolism (Gmps, Oat) and mitochondrial metabolism (Acaa1a and Sdhb). At 24 hpi (**Figure 8D**), the exclusive protein network displayed greater functional cohesion, centered on immunological processes. Interactions were observed among proteins involved in the inflammatory response (Hmox1 and Cp), leukocyte migration (Csf3 and Cxcl2), mitochondrial modulation (Mtco2, Ndufs3, Phb2) and cellular stress response (Tnfaip2 and Dnm1; **Supplementary Figure S2**). Functional enrichment analysis showed that at 8 hpi (**Figure 9A**), the exclusive proteins were mainly associated with terms such as cell cycle checkpoints, innate immune system, and metabolic process. In contrast, at 24 hpi (**Figure 9B**), there was a strong enrichment of immune-related processes, including immune system process and negative regulation of signal transduction. As a final step, we explored whether exosomes also carry pathogen-derived proteins, which may represent a potential mechanism of direct host-pathogen communication.

### 3.7 Identification of bacterial broteins in exosomes

Analyzing the proteome of exosomes secreted by macrophages infected with *B. abortus*, 17 and 10 *B. abortus* proteins were identified at 8 hpi and 24 hpi, respectively. These proteins were present in all three biological replicates (**Table 1**). Of these proteins, five were shared across both infection time points: AtpA, AtpD, BAB1_0238, groEL, and SodC. The subcellular localization of these proteins in the bacteria was predicted using the PSORTb server. At 8 hpi, proteins originating mainly from the cytoplasm, outer membrane, and periplasm were observed, while at 24 h, proteins located mainly in the cytoplasm and periplasm were identified (**Table 1**). On the other hand, no proteins with homology to *B. abortus* were identified in the EXOC proteome.

**Table 1:** *B. abortus* proteins identified in exosomes derived from infected macrophages.

ID	Protein name	Post-infection incubation time (h)		Subcellular localization
		8	24	
Q2YLI5	ATP synthase subunit alpha	**✓**	**✓**	Cytoplasmic
Q2YLE6	ATP synthase subunit beta	**✓**	**✓**	Cytoplasmic Membrane
Q2YP67	Bacterial extracellular solute-binding protein, family 1	**✓**	**✓**	Unknown
Q2YMJ1	Modulator of DNA gyrase	**✓**		Cytoplasmic
Q2YRQ2	Outer membrane protein assembly factor BamA	**✓**		Outer Membrane
Q2YLP2	Chaperone protein ClpB	**✓**		Cytoplasmic
Q2YQV2	Chaperone protein DnaK	**✓**		Cytoplasmic
Q2YIJ3	Chaperonin GroEL	**✓**	**✓**	Cytoplasmic
Q2YIJ2	Co-chaperonin GroES	**✓**		Cytoplasmic
Q2YLR6	Outer membrane lipoprotein Omp19	**✓**		Outer Membrane
Q2YMY7	Porin Omp2b	**✓**		Outer Membrane
Q2YQH3	Peptidoglycan-associated lipoprotein	**✓**		Outer Membrane
Q2YIY2	Periplasmic binding protein/LacI transcriptional regulator	**✓**		Periplasmic
Q2YKV9	Superoxide dismutase [Cu-Zn]	**✓**	**✓**	Periplasmic
Q2YRD7	Tol-Pal system protein TolB	**✓**		Periplasmic
Q2YM08	Elongation factor Tu	**✓**		Cytoplasmic
Q2YPK5	Invasion protein B homolog BAB1_0368	**✓**		Unknown
Q2YRV0	Isocitrate dehydrogenase [NADP]		**✓**	Cytoplasmic
Q2YK12	DUF1269 domain-containing protein		**✓**	Unknown
Q2YKI4	Bacterioferritin		**✓**	Cytoplasmic
Q2YK18	Probable acid stress chaperone HdeA		**✓**	Unknown
Q2YLS0	Succinate-CoA ligase [ADP-forming] subunit beta		**✓**	Cytoplasmic

*B. abortus* proteins at 8 and 24 hpi described according to the Uniprot database (ID), name, and predicted subcellular localization.

## 4 Discussion

The interaction between intracellular pathogens and immune cells establishes a dynamic adaptation scenario (53). In this context, pathogens develop multiple strategies to block or interfere with critical points of the host’s immune response, thus ensuring their intracellular persistence (54). Several studies have shown that exosomes, a subpopulation of extracellular vesicles (EVs), play a fundamental role in intercellular communication, modulating processes such as inflammation, cell differentiation, and immune cell activation (33, 55, 56). Analyzing the molecular content of exosomes released by macrophages infected with *B. abortus* therefore represents a relevant avenue for understanding the mechanisms of immune evasion and manipulation induced by this pathogen in infected cells. Although there is growing interest in the role of exosomes in the context of infectious diseases, their specific characterization during brucellosis remains largely unexplored. Available evidence shows that exosomes derived from macrophages infected with *B. melitensis* can reduce the intracellular survival of the bacteria in immune cells (38). Furthermore, these exosomes have been shown to transport bacterial antigens capable of influencing the polarization of uninfected macrophages, favoring a proinflammatory M1-type response (36). However, the detailed proteomic characterization of exosomes secreted by macrophages during *B. abortus* infection has not been addressed in depth. Thus, in this study we performed a comparative proteomic analysis of exosomes derived from RAW 264.7 macrophages infected with *B. abortus*, at early and late times post-infection (8 hpi and 24 hpi), with the aim of recognizing proteins that allow identifying potential immunoregulatory mechanisms triggered by this pathogen through the release of extracellular vesicles.

Initial characterization confirmed that exosomes isolated under all experimental conditions used in this work met the classic criteria of size and morphology, in accordance with those described by Théry et al. (2018), thus validating their origin, isolation, and purity. In addition, the consistent detection of classical exosomal markers such as CD9, CD63, and CD82 across all analyzed samples further confirming that the vesicles obtained correspond to exosomes. At a quantitative level, an increase in the number of proteins identified in exosomes derived from infected macrophages (EXOI) was observed at 8 hpi, while at 24 hpi the total number of proteins identified was similar between infected and control macrophages. This finding suggests a dynamic protein profile, where the exosomal protein load varies depending on the stage of infection (57). Notably, these changes were not associated with morphological or size alterations in the vesicles, suggesting a specific reorganization of the protein content of exosomes rather than a nonspecific activation of their biogenesis. Subcellular localization analysis showed that the majority of exosomal proteins originate from the cytoplasm and the nucleus, in line with what has been previously described in proteomic characterization studies of exosomes (24, 58). However, a sustained presence of proteins associated with intracellular membranes derived from the endoplasmic reticulum (ER) and mitochondria was also observed, which could reflect an active recruitment of proteins linked to metabolism and protein processing, processes tightly regulated during *Brucella* infection (14, 15, 59).

Analysis of differentially expressed proteins (DEPs) showed distinct patterns depending on the time post-infection. At 8 hpi, EXOI exosomes overexpressed proteins related to protein biosynthesis, energy metabolism, and ER processing, consistent with a state of early metabolic activation of the macrophage (60). This activation may reflect an initial response to infection, oriented towards the production of inflammatory mediators, receptors, and other immunomodulatory molecules (61). Importantly, early metabolic reprogramming in macrophages has been shown to indirectly condition immune responses by regulating the availability of energy and key metabolites required for cytokines synthesis and by shaping the cellular redox state that drives immune polarization (62, 63) In parallel, underexpression of proteins associated with lysosomes, proteasome, and antigen presentation was observed, which may suggest a potential manipulation of these pathways by *B. abortus* in favor of its intracellular persistence. At 24 hpi, the proteomic profile of EXOI exosomes showed a functional reorientation towards mitochondrial and oxidative stress pathways, accompanied by a sustained decrease in antigen processing, endocytosis, and apoptosis pathways. This pathogen has been described as capable of interfering with phagosome maturation, preventing its fusion with the lysosome, and blocking antigen presentation via MHC I and II, through mechanisms that include the modulation of TLRs and the secretion of TIR-type effector proteins such as TcpB (14, 64, 65). These changes could reflect a macrophage adaptation to a state of persistent infection, potentially involving redox regulatory mechanisms, while coinciding with a suppressed processes related to antigen presentation and cell elimination, thereby potentially contributing to immune evasion by *B. abortus* (66).

A particularly interesting finding was the identification of exclusive proteins in EXOI exosomes, which may reflect a shift in functional enrichment from metabolic and nuclear processes toward immune-related functions at the later time point of infection. At 8 hpi, EXOI-exclusive proteins clustered into functional categories related to ribonucleoprotein RNA processing (Snu13, Trmt112, Rsl1d1, Mrto4), retromer-mediated endosomal trafficking (Snx1, Snx6, Igf2r), and cellular metabolism (Gmps, Oat, Acaa1a). The exclusive presence of retromer proteins in exosomes derived from infected macrophages could reflect a functional reprogramming of endosomal trafficking potentially associated with *B. abortus* infection. Under basal conditions, the retromer system is involved in the retrograde recycling of proteins from endosomes to the Golgi apparatus or the plasma membrane, preventing their lysosomal degradation and promoting their recycling (67). However, during infection, macrophages intensely activate endosomal pathways as part of their early response to process and eliminate the intracellular pathogen (68). This activation could favor the recruitment of retromer components to endosomal compartments that may become incorporated into multivesicular body formation, facilitating their selective inclusion in exosomes. Alternatively, the manipulation of vesicular trafficking by *B. abortus*-aimed at preventing phagosomal maturation and favoring the establishment of the replicative niche, might also influence the fate of these proteins, potentially diverting them from the degradative route toward the exosomal pathway (16, 69). Thus, the detection of Snx1, Snx6, and Igf2r exclusively in exosomes from infected macrophages may reflect alterations in endosomal trafficking, and may represent a mechanism through which the pathogen modulates host vesicle composition, potentially contributing to immune evasion.

On the other hand, at 24 hpi, the exclusive protein repertoire of EXOI exosomes may reflect a shift in enrichment toward proteins associated with immunological processes. Functional analysis suggested an enrichment in terms linked to the immune system, negative regulation of signal transduction and inflammatory signaling. Proteins such as Csf3, Cxcl2, Hmox1, Gsdmd and Ifi35 were identified, which have previously been associated with the inflammatory response, leukocyte recruitment and in some contexts the activation of immunogenic cell death pathways such as pyroptosis (70–73). In particular, GSDMD acts as a key effector in pore formation during pyroptosis, allowing the release of proinflammatory cytokines such as IL-1β (71, 74). The presence of this type of proteins in exosomes may suggest a potential signaling role, through which infected macrophages might contribute to the amplification or dissemination of immune activation cues within the tissue microenvironment. In this context, the detection of CSF3 and IFI35 is noteworthy, both molecules have been previously described as having immunomodulatory potential on neighboring cells. CSF3, as a hematopoietic cytokine, can stimulate neutrophil differentiation and migration (75), while IFI35, characterized as a DAMP protein, can induce TLR4 activation and the production of proinflammatory cytokines (73). Their incorporation into exosomes may suggests that these vesicles could act as potential mediators for intercellular communication during infection. Previous studies have shown that exosomes derived from macrophages infected with *B. melitensis* can induce M1 polarization and promote protective responses mediated by cytokines such as TNF-α and IL-12 (36), reinforcing the potential immunomodulatory role of exosomes during brucellosis.

Bacterial proteins were consistently detected in exosomes derived from macrophages infected suggesting that their incorporation may be a common feature of *B. abortus* infection. Specifically, GroEL and SodC were present at both 8- and 24-hours post-infection, whereas Omp19, Omp2b, DnaK, and the invasion protein B homolog BAB1_0368 were detected only in the early phase (8 hours). This profile may reflect a temporal pattern in bacterial protein release, where early-phase proteins are associated with invasion and microenvironment modulation. For example, Omp19 has been reported to inhibit lysosomal proteases and protects bacterial antigens (76, 77); Omp2b, has been implicated in modulating permeability and the innate immune response (78, 79); DnaK is a cytoplasmic chaperone involved in protein folding under stress conditions (80–82); and BAB1_0368, which shares sequence similarity with known virulence factors involved in invasion and intracellular niche remodeling (83, 84), may play a role in *Brucella* pathogenesis. In contrast, the sustained presence of GroEL and SodC may indicate a potential role in supporting bacterial persistence and immune evasion. Notably, both proteins are among the main immunogenic components during *Brucella* infection (85), where GroEL has been shown to promote the production of TNF-α and IL-6 in macrophages and to induce a Th1-type immune response in mice (86, 87). Meanwhile, SodC contributes to bacterial survival by neutralizing superoxide anions, thereby protecting Brucella from the phagocyte respiratory burst (88). These findings reinforce the notion that exosomes may not be mere cellular byproducts, but could function as potential mediators of pathogen-host communication. Their early phase protein content may be associated with invasion-related processes, while their later profiles may reflect features linked to immune modulation and persistence of *B. abortus*. Subcellular localization analysis showed that most of the bacterial proteins identified in exosomes corresponded to cytoplasmic components, and this trend was maintained both at 8 and 24 hpi. The detection of intracellular proteins such as GroEL, DnaK and SodC in exosomes suggests that their incorporation could occur after partial lysis of phagocytosed bacteria or possibly via active secretion mechanisms not yet characterized, occurring during the formation of multivesicular bodies. Previous studies in other intracellular pathogens have shown that bacterial proteins can access the host cytosol through secretion systems such as SecA or type VII, and subsequently be targeted to exosomes via post-translational modifications like mono-ubiquitination, as well as through ESCRT or clathrin-dependent pathways (89). These observations support the possibility that *B. abortus* may exploit similar mechanisms to facilitate the incorporation of its proteins into exosomes derived from macrophages. This observation suggests that subcellular localization may not limit their incorporation to extracellular vesicles, which support the hypothesis that exosomes derived from infected macrophages could act as vehicles for PAMPs and potentially contribute to activation of immune signaling or host tolerance (90). Similar findings have been reported in other intracellular pathogens. For example, exosomes from macrophages infected with *Mycobacterium tuberculosis* contain bacterial proteins that can activate immune responses (34, 91–93), In *Salmonella enterica* infection, exosomes transport bacterial components that modulate host signaling (35, 94), while in *Listeria monocytogenes* infection, they can even carry bacterial DNA capable of activating the cGAS-STING pathway, a spotent trigger of innate immunity (95, 96). Thus, the sustained presence of these *B. abortus* proteins in exosomes supports the idea that these vesicles may not only reflect the host cell’s infection status, but could also function as potential mediator of pathogen-host communication with implications for immune modulation. Taken together, these observations underscore the “double-edged sword” nature of exosomes, which may simultaneously promote immune activation while facilitating immune evasion and bacterial persistence.

Notably, our study also suggests that *B. abortus* proteins are consistently incorporated into exosomes in a temporally regulated manner, providing a novel perspective on their potential role in host–pathogen communication.

To validate the immunomodulatory potential of exosomes derived from *B. abortus*–infected macrophages, future studies should include functional assays aimed at dissecting their impact on host immune responses. A central proposed experiment would involve the preconditioning of unstimulated macrophages with isolated exosomes prior to infection, followed by quantification of bacterial replication and host responses. These responses could be evaluated by assessing the expression of inflammatory cytokines (e.g., TNF-α, IL-6, IL-10, IL-12) through qRT-PCR and protein secretion via ELISA or multiplex assays.

This approach would not only provide insight into the biological consequences of exosome-mediated communication, but also establish a functional bridge between the proteomic landscape described here and downstream immune outcomes, laying the groundwork for future *in vitro* and, if feasible, *in vivo* studies.

## 5 Conclusion

Taken together, the results described here suggest that exosomes derived from macrophages infected with *B. abortus* not only reflect the altered functional status of the host cell, but may also contribute to the propagation of immunomodulatory signals that could facilitate bacterial infection and persistence. These observations support the hypothesis that exosomes may act as mediators at the pathogen-host interface, rather than being mere cellular byproducts. Furthermore, their characteristics make them promising candidates for the development of infection biomarkers and potential therapeutic or vaccine tools (39, 97–99).

## Data Availability

The datasets presented in this study can be found in online repositories. The names of the repository/repositories and accession number(s) can be found in the article/**Supplementary Material**.
