# Supplementary material for: Proteomic analysis of exosomes from Brucella abortus-infected macrophages reveals possible mechanisms of immune evasion and host modulation
Source: Front Immunol. 2025 Oct 30;16:1685245. doi: 10.3389/fimmu.2025.1685245 (PMC12611657; doi:10.3389/fimmu.2025.1685245)
Supplement: Supplementary file 1 [file DataSheet1.pdf]

Supplementary Table 1. Detection of the exosomal markers CD9, CD63, and CD82 by proteomics at 8h and 24h in exosomes released by macrophages infected and non-infected with *Brucella abortus* 2308.

Marker	UniProt	Time (h)	Condition	Replicate	Normalized intensity	Unique Peptides
CD9	P40240	8	Non-infected	1	1.17819E-07	4
				2	4.57258E-09	4
				3	2.01823E-08	3
			Infected	1	7.90815E-09	4
				2	3.89374E-08	4
				3	4.47875E-09	4
		24	Non-infected	1	0.078010639	9
				2	3.82666E-08	7
				3	5.50788E-08	8
			Infected	1	0.055351319	9
				2	3.92844E-08	5
				3	6.56541E-08	8
CD63	P41731	8	Non-infected	1	6.062E-08	2
				2	2.61703E-08	2
				3	7.33909E-09	2
			Infected	1	3.38579E-08	2
				2	1.86562E-08	2
				3	2.64281E-08	2
		24	Non-infected	1	2.35416E-08	3
				2	4.0069E-09	2
				3	7.24996E-09	3
			Infected	1	2.00974E-08	4
				2	2.33186E-08	2
				3	1.21952E-09	3
CD82	P40237	8	Non-infected	1	5.72644E-08	5
				2	2.21698E-08	5
				3	8.59461E-08	4
			Infected	1	1.93382E-08	5
				2	1.29023E-08	5
				3	2.27585E-08	5
		24	Non-infected	1	3.30761E-08	7
				2	2.14132E-08	7
				3	2.52673E-08	7
			Infected	1	6.19413E-09	7
				2	9.61812E-10	6
				3	5.72065E-09	7

Normalized intensity values of exosomal markers CD9, CD63, and CD82 detected by proteomics in exosomes released by macrophages either infected or non-infected with *Brucella abortus* 2308 at 8 h and 24 h. Each condition was analyzed in triplicate (n = 3). Proteins were considered confidently identified when supported by ≥2 unique peptides and a protein-level FDR < 1%.

Supplementary Table 2. Differentially expressed proteins (DEPs) identified at 8 h in exosomes released by macrophages infected with *Brucella abortus* 2308 compared to non-infected controls.

Protein ID	logFC	P_value
P62082	0.631916862	0.042422629
P56480	0.650765335	0.029219081
P63260	0.671713049	0.038393945
Q99MN9	0.699402161	0.030423637
Q91V41	0.702768691	0.040384727
Q00623	0.713173747	0.027036595
Q3U1J4	0.737416591	0.028825438
P24668	0.778480263	0.024498673
P61161	0.784066188	0.035372356
P46638	0.801256858	0.024796043
P63325	0.815723695	0.041725063
P80313	0.82056941	0.026310231
Q9QUI0	0.829837956	0.016013454
Q8BU30	0.831552731	0.048595184
Q9D1A2	0.839978261	0.041027043
O08810	0.845050846	0.036647046
P97384	0.853334474	0.02411751
Q01853	0.871334705	0.020697141
P62849	0.87849151	0.030280269
P46471	0.881177756	0.033361899
P58252	0.890182035	0.012763277
Q02053	0.892263884	0.028757483
Q922B2	0.898891687	0.041126327
Q8BT60	0.900434854	0.006228671
P0C0A3	0.934594475	0.032563369
Q60634	0.952985081	0.018186451
Q8BIJ6	0.956601294	0.039695013
P35564	0.960226538	0.01113015
P51174	0.961960363	0.022373192
P54071	0.964998148	0.049777133
Q91V92	1.002251737	0.030519062
P59108	1.015567861	0.00676201
P21956	1.02205144	0.003998998
P62317	1.026086854	0.042909256
Q99MD9	1.031671638	0.013968355
Q9JKB3	1.033421016	0.022222064
P24270	1.033502425	0.013077874
P63038	1.03516707	0.034278104
Q9Z1D1	1.040191639	0.026886704
P97429	1.048800545	0.027666816
P49710	1.049118606	0.029656372
Q01339	1.050675565	0.030729402
Q9D6R2	1.053385306	0.046283037
Q9CZX8	1.057419	0.04237559
P97461	1.058299826	0.014231039
Q69ZN7	1.08864381	0.022925927
Q8C166	1.090959831	0.015186158
Q8VBT6	1.092405536	0.023314694
Q91W89	1.094203209	0.042303043

Q8BGQ7	1.121637067	0.008882412
O35474	1.132280859	0.006132638
Q9Z1F9	1.137673474	0.019968086
P20029	1.141958316	0.034058523
Q9QYB1	1.152104997	0.024977016
P62852	1.157567262	0.012780382
Q8K2T8	1.159822138	0.030043174
P63276	1.181356419	0.013298042
B2RQC6	1.186723487	0.044180747
P19096	1.187126443	0.003762194
Q9WUM4	1.23033931	0.048191091
Q9CQX2	1.234154931	0.00381492
P27870	1.238165145	0.045913405
P35293	1.242869441	0.006109098
O54833	1.249076904	0.028446487
P51863	1.252740033	0.024377811
Q9DB05	1.287677181	0.038197144
Q64514	1.3097649	0.015440814
Q8BSY0	1.310039953	0.022343105
Q922J3	1.331916582	0.033639482
E9Q5C9	1.338232389	0.013907215
P70168	1.348418439	0.004337719
P08003	1.365292785	0.027746553
Q9DCX2	1.368868693	0.010114206
P14824	1.378425747	0.032826094
Q8BLN5	1.396244962	0.001237039
P31230	1.398000636	0.022896999
Q9R0P3	1.40052581	0.007631971
P62192	1.406311923	0.046867871
P56395	1.427814379	0.011606007
P62305	1.434562469	0.004709817
P97855	1.448461103	0.02754623
Q9QZE5	1.450310441	0.03708786
P61021	1.462062483	0.004868178
P57776	1.476522707	0.030229215
Q60737	1.477824102	0.047893827
P07356	1.484147058	0.000324842
P62821	1.494941954	0.001744954
Q91Z22	1.554987773	0.041922744
P45952	1.555302256	0.012319606
Q9ERK4	1.557286376	0.032141802
O35226	1.59445945	0.0171088
Q61074	1.595564399	0.044963033
P67871	1.613271149	0.010220454
Q9DBE9	1.616085816	0.011494628
P62843	1.656163349	0.013590074
Q8N7N5	1.662405021	0.002836511
Q8R317	1.670030828	0.0133776
Q8VEH3	1.670434294	0.002709654
P62900	1.69859047	0.003774312
Q9D1G1	1.736670319	0.002714555
Q9CZ44	1.777035615	0.033060139
O55126	1.831841731	0.000951321
Q9QUM4	1.83352357	0.00330905
Q07076	1.853312107	0.001254186
Q9R1J0	1.871775067	0.014102648
Q3THE2	1.872155526	0.002445434

Q99L13	1.884498782	0.014716193
Q9DCW4	1.886089952	0.035927741
Q8BTJ4	1.889430383	0.004504854
Q921G7	1.925439321	0.000170434
P47856	1.986183609	0.002176094
P17918	1.986544395	0.00241033
Q64337	1.992978451	0.000854266
P48999	2.015424275	0.013049363
Q62087	2.07645146	0.001060235
O35639	2.085046601	0.001782623
O54984	2.114113061	0.012934332
Q6ZWV7	2.143979181	0.004874987
P81117	2.149314602	0.042597549
Q9D0F3	2.206244626	0.009372322
Q59J78	2.321218647	0.000451016
Q0VGB7	2.351476947	0.012712919
P10107	2.355861825	8.87341E-05
Q6PDI5	2.366622508	0.002572665
Q6PB66	2.394213324	0.036103726
Q91ZA3	2.41964602	0.00946472
Q9CQW2	2.49494342	0.000144294
Q9EPJ9	2.562739218	0.00069066
Q9CYZ2	2.667658332	7.63911E-05
Q62393	2.775362208	4.85382E-05
O70251	2.819878815	0.004784697
O55125	2.867562732	0.000519328
Q9DCN2	2.86942268	0.000418363
Q9DBG5	2.952115888	0.001172422
Q9Z2I8	3.427878401	0.000892553
Q7TMM9	-3.96753323	0.002009433
Q9Z320	-3.409425371	0.013771089
P02535	-3.279549017	0.010396021
Q61414	-3.262723478	0.013897211
P07310	-2.904946025	0.007426665
Q921I1	-2.871443876	0.001651168
P08730	-2.677609985	0.02873698
Q9EPL2	-2.483037886	0.013057585
Q8BFR4	-2.416220921	0.010013259
Q921M3	-2.313846444	6.43721E-05
Q8K411	-2.310105978	0.015913176
P09581	-2.236180426	0.000654358
Q9D952	-2.217156208	0.010755205
Q99KN1	-2.18850076	0.022684978
O89017	-2.105952596	0.017682277
Q9Z331	-2.092942796	0.04417289
P06797	-2.076911723	0.02432425
P19001	-2.049697515	0.009047192
O08848	-2.022934567	0.022025573
Q6ZQ38	-2.011338096	0.029126269
P55012	-1.992293197	0.01240221
Q9QZC7	-1.962905972	0.010768188
Q8R0J7	-1.95130667	0.002829517
Q3UH93	-1.926200574	0.034958419
P27641	-1.920418818	0.031182839
Q9R0H5	-1.911193187	0.014580983
Q9QXS1	-1.899571716	0.001256903
Q64374	-1.897266236	0.004505963

Q9CZ28	-1.896277107	0.016117891
O35640	-1.891939088	0.032779555
Q811D0	-1.889482636	0.021084849
P15105	-1.889109311	0.037131394
Q8CB27	-1.853808378	0.040127481
P11680	-1.831854395	0.012149861
Q66JY6	-1.812761273	0.01609014
A1L317	-1.774779857	0.046896147
Q6ZQ93	-1.770102884	0.048271162
Q6IFZ6	-1.760371501	0.000726525
Q9CQ26	-1.727458242	0.005257455
Q9D176	-1.727278614	0.001142269
P25118	-1.664061713	0.005817939
P10605	-1.648915304	0.00176624
Q6URW6	-1.643440497	0.037703882
Q8R366	-1.638824545	0.00486956
P36895	-1.637833557	0.040339274
Q3UH60	-1.635107034	0.005616421
Q9R269	-1.612094786	0.016873417
Q3TH73	-1.598205339	0.000915926
P51855	-1.596106671	0.017540776
Q8CGR7	-1.568427064	0.002866073
P01632	-1.567903504	0.012369168
Q61781	-1.567439987	0.007317056
P35762	-1.557785242	0.000474991
Q07797	-1.545470065	0.014234296
Q6PGL7	-1.530692604	0.043301401
Q50L42	-1.518240145	0.024139882
O35350	-1.51772878	0.042342935
Q6KAU4	-1.515695431	0.010205708
Q61187	-1.498486168	0.001231421
P21550	-1.480273938	0.027536941
Q99PS0	-1.461980173	0.037867193
Q9WUU7	-1.450104296	0.016866363
P35492	-1.42939443	0.013244738
Q9QZ08	-1.42714008	0.029910439
Q0VBK2	-1.422060746	0.021480434
O35598	-1.411342635	0.001457946
Q8R016	-1.409032014	0.008747137
Q8R105	-1.403999871	0.003296945
P02301	-1.397732803	0.008533411
Q8CDG5	-1.3966103	0.031749861
P12388	-1.393987895	0.034161991
Q8BI08	-1.393644061	0.023525363
Q9D708	-1.376912925	0.031777244
Q9EST1	-1.355791585	0.013717661
Q6P5F7	-1.353942942	0.007708386
Q61470	-1.346652067	0.011855977
Q9JLF6	-1.337157242	0.016915457
Q9WV07	-1.330015564	0.017956908
Q9WTL4	-1.317818497	0.037424392
Q2VIS4	-1.309639865	0.039027307
Q8BGZ7	-1.299251168	0.035413966
Q9R111	-1.293191423	0.032182301
Q00651	-1.289218995	0.00161097
O35516	-1.283104721	0.041247512
P70124	-1.280855922	0.036840886

Q9Z2K1	-1.26860815	0.002753374
Q8BNQ3	-1.264689562	0.009370816
Q9DAS9	-1.264025582	0.034448096
Q61176	-1.259330023	0.021556092
Q9WU78	-1.257237085	0.006885922
Q922U2	-1.257137785	0.001657789
P97820	-1.252538918	0.008396539
P09055	-1.235501613	0.003558939
P12265	-1.234308481	0.021873458
Q3TD16	-1.224917317	0.03086336
O09159	-1.210310128	0.037649422
Q9JKB1	-1.201299405	0.040575416
Q91VW3	-1.200128154	0.037901147
P18242	-1.169941912	0.007037377
Q9WU81	-1.169214372	0.022233115
P10853	-1.166888936	0.005618476
P97350	-1.159832213	0.030748279
Q9D7X8	-1.14250033	0.04630159
O08992	-1.134431156	0.009013827
P62806	-1.132710786	0.004616851
P40237	-1.113030841	0.004979607
Q64523	-1.107302752	0.037414572
C0HKE1	-1.100804539	0.007906485
P02468	-1.098755892	0.03914942
P17563	-1.093088498	0.04465733
Q08189	-1.091999051	0.034702704
Q9ET30	-1.062458718	0.044049724
Q8VDD5	-1.06000432	0.020818263
Q9QZ06	-1.050223749	0.049333575
P30993	-1.048422788	0.024877672
Q61495	-1.04065338	0.027875587
Q61703	-1.037906306	0.023591183
Q61598	-1.028218335	0.032187636
Q61235	-1.026176206	0.020424938
O55234	-0.997828013	0.041438993
P10923	-0.995599713	0.038567069
E9Q557	-0.982472901	0.037495476
P97298	-0.977276279	0.026243826
Q6IME9	-0.975819084	0.027487976
P99026	-0.972464174	0.043937728
O70582	-0.97106205	0.043384209
P0C6B7	-0.958227793	0.027828948
O70435	-0.953467699	0.044776098
Q8QZY6	-0.938088756	0.029193321
Q99P72	-0.930156586	0.036684869
P01898	-0.928819515	0.039620694
Q9EPK7	-0.92097541	0.044015139
P30204	-0.919215788	0.033770269
Q64727	-0.807440974	0.03297761
P63037	-0.796186819	0.041509052
P10404	-0.763759374	0.021796306
P62983	-0.721035637	0.016651067

Proteins differentially expressed between exosomes from infected and non-infected macrophages at 8 h are listed. logFC values represent log2 fold changes, where positive

values indicate proteins upregulated in infected samples and negative values indicate proteins downregulated in infected samples (relative to controls). Statistical significance was determined using Student's t-test, and p-values are shown for each protein. Proteins with $p < 0.05$ were considered significant DEPs.

Supplementary Table 3. Differentially expressed proteins (DEPs) identified at 24 h in exosomes released by macrophages infected with *Brucella abortus* 2308 compared to non-infected controls.

Protein ID	logFC	P_value
Q3U7R1	0.572096709	0.035828067
Q91ZN5	0.573772957	0.036520361
Q07797	0.753276953	0.042493793
Q01320	0.895576724	0.033175712
Q62159	1.001929322	0.030387413
Q8R5J9	1.097498977	0.03785182
A2AQP0	1.107471096	0.034458299
Q8C4Q6	1.111970721	0.03117123
Q60902	1.288676651	0.027335977
P25085	1.320360825	0.026915766
Q8BTW3	1.638525741	0.033510884
Q9D8S9	1.844245521	0.036288264
P08122	1.908189538	0.033821472
Q99JR5	2.039755138	0.034371406
P56959	2.052353052	0.02719635
Q62384	2.263552993	0.031892157
P13379	5.057828128	0.032581374
Q8BLN5	5.093056959	0.032158999
P39688	5.35155416	0.031925212
P97429	5.379287595	0.011314207
Q9CQF9	5.492143796	0.026200861
P25976	5.581170225	0.029061278
Q99JX4	5.627592236	0.028320215
Q8R326	5.649592995	0.028744833
Q61249	5.694374509	0.030933922
O70310	5.717254649	0.014379543
Q8QZT1	5.734190208	0.029848135
Q91WK2	5.736649511	0.026599472
Q61152	5.853056232	0.029536029
O35864	5.880327375	0.020300784
Q8VCT3	5.888218732	0.027068254
Q3UUG6	6.019152326	0.021911565
Q8K2B3	6.077728577	0.024484276
E9Q7G0	6.089303387	0.028719524
P61202	6.097345466	0.02742277
P43024	6.099619574	0.02826779
P04223	6.132594203	0.021498272
Q9D083	6.183853462	0.026684831
A1L3P4	6.185110207	0.025877264
A2AJ15	6.225079262	0.020380663
P27600	6.254907968	0.017561569
P02535	6.283206939	0.020904315
P06804	6.290028782	0.021374609
Q9JLV1	6.29740408	0.021650956
Q3UI43	6.298386417	0.022184865

Q8CI94	6.305055181	0.024733296
Q9CZ13	6.329551398	0.015308894
P63082	6.3379396	0.020602953
Q9R0Q8	6.349903841	0.021996758
Q9JJH1	6.427704817	0.02008305
P97457	6.456745075	0.020314601
P97310	6.457694254	0.0203169
Q8VI75	6.481957342	0.014299257
Q8K201	6.484772319	0.017354237
Q9D1R9	6.488504154	0.018351673
P21440	6.507901292	0.017757539
Q9D7X8	6.511937476	0.01606013
Q9CQQ7	6.52655744	0.013536839
O08579	6.538276483	0.01175574
P63328	6.567487925	0.013195937
Q8BL66	6.595961637	0.007049846
Q9Z130	6.626572327	0.008840317
P60202	6.631006862	0.008465891
P19157	6.65491161	0.00583909
P10922	6.660536652	0.00589387
Q5SUS0	6.661596825	0.00550715
P70658	6.668451552	0.003967119
P07141	6.759367574	0.002157122
P52875	6.775879765	0.000704029
P47968	6.79302633	0.000666291
O70133	6.811894972	0.00053376
P23336	6.865507277	0.000509128
Q9CQV6	6.939875339	0.000333568
Q8BGQ6	7.000256953	0.000181286
Q9JI75	7.134570319	0.000176059
Q7TMF5	7.24324544	4.65293E-05
P00405	7.417599899	0.000124939
Q91ZJ9	7.561249891	3.52947E-05
P51569	7.779025215	2.8479E-05
P70699	7.85674691	2.92954E-05
Q8CGF7	7.947741717	2.56678E-05
Q9CYA0	8.092818133	6.79756E-06
Q91VD9	8.267963757	5.35427E-06
Q9CWW7	8.314127979	1.38329E-06
Q8VBV7	8.586424524	3.40829E-06
Q5SSZ5	8.996972124	9.14459E-07
P56135	9.069544622	1.67761E-06
Q8C5L6	9.075176179	3.35371E-07
P97390	9.102625544	1.00384E-06
P41241	9.385765127	9.33323E-07
Q8K3H0	9.818031824	3.69495E-07
Q8K310	9.898092413	2.30239E-07
Q8R146	9.904171325	3.62491E-07
Q8BGT0	9.92356103	1.45816E-07
Q9ERG2	10.00273768	6.70825E-08
P45377	10.61399254	1.84399E-08
Q8K4Z3	-12.4988858	0.040200979

Q62048	-10.8021964	0.038638229
P31938	-10.44249033	0.029059934
Q9Z0M5	-9.656079243	0.035247022
Q9CQV4	-9.46923318	0.037572286
O08759	-9.428270104	0.044669753
P97492	-9.15019145	0.046659491
Q9JL56	-9.084813928	0.038368488
Q9DD03	-9.066791646	0.046298247
Q8CB77	-8.615120987	0.042983938
Q6PIE5	-8.527685362	0.037881464
Q6PIU9	-8.220697962	0.046009395
Q8K1Z0	-7.983646626	0.046955824
Q9D0M3	-7.818892645	0.037697707
Q32M21	-7.715728843	0.043131872
Q9Z1R3	-7.512689461	0.044948782
O70281	-7.455354317	0.041618802
O35682	-7.435261538	0.041838715
P53564	-7.412331873	0.036621349
P0C8B4	-7.277030793	0.043664433
Q6P8I4	-7.222219887	0.041809228
Q6U7R4	-7.119110836	0.04935592
O55234	-7.114213848	0.039360101
Q9Z0X1	-6.980457994	0.046307007
P39054	-6.954052061	0.02491856
Q5SYH2	-6.922476914	0.041880951
Q3THG9	-6.884779059	0.043299207
Q9D855	-6.834222412	0.026322784
Q9R1S8	-6.793045395	0.040393848
P01942	-6.762526933	0.049930501
Q6ZQ38	-6.754510981	0.022768163
Q9ES00	-6.678843275	0.036355705
P45952	-6.663871871	0.036392628
P14576	-6.583012694	0.038868096
P56376	-6.570548585	0.042344106
Q9QY33	-6.543574138	0.041045177
Q9CQW1	-6.528448919	0.029557283
Q8K2C7	-6.493165606	0.039600853
Q9Z2H5	-6.474272379	0.048856577
P49282	-6.472004528	0.049770479
P61967	-6.42710678	0.029753286
O55143	-6.389514524	0.049823361
Q62165	-6.357228358	0.0405733
P47791	-6.297791858	0.044985553
O88696	-6.297499015	0.046575425
Q3U497	-6.285312712	0.04780809
P47964	-6.266565884	0.038394497
Q8CJ61	-6.231552367	0.029304623
Q8K211	-6.207794127	0.047016279
Q9DC37	-6.133917303	0.036905966
Q6P1B3	-6.131714508	0.045994091
Q99MR6	-6.087191816	0.043743548
P62080	-6.073176479	0.031298518

Q5H8C4	-6.071698812	0.04618494
Q99N07	-6.017544226	0.038304252
P00375	-5.960690476	0.038518277
Q9R1P3	-5.851042666	0.032732825
Q91WB7	-5.72784686	0.047898735
P01899	-5.716767017	0.041181507
P97369	-5.698671498	0.044256811
Q99LE1	-5.669449373	0.036542717
P01898	-5.610843909	0.040562566
Q1HFZ0	-5.589055895	0.044418158
Q921I1	-5.580014609	0.039237741
Q5FWK3	-5.571293196	0.04343854
Q8R2U4	-5.51587523	0.032286275
O35664	-5.509150442	0.026298304
Q9DBU0	-5.413276412	0.037959245
Q62376	-5.245176918	0.043543755
Q8BTI8	-5.115916993	0.039109815
Q8BMP6	-5.000351703	0.039361251
Q9ESY9	-2.657498348	0.036512724
P58021	-2.18104854	0.04048211
Q9D8W5	-1.859746287	0.040522245
Q8BHA3	-1.622786458	0.036211263
Q64327	-1.57105258	0.030565361
Q8VEH3	-1.16756654	0.026403918
P48722	-1.137823644	0.039260206
O70274	-1.11652874	0.040027786
P97821	-1.069006	0.039229597
Q64213	-1.045118313	0.038360647
P70697	-0.921112693	0.04053179
Q9QVP9	-0.69437301	0.036439404
Q8BGE4	-0.555767367	0.039882481

Proteins differentially expressed between exosomes from infected and non-infected macrophages at 24 h are listed. logFC values represent log2 fold changes, where positive values indicate proteins upregulated in infected samples and negative values indicate proteins downregulated in infected samples (relative to controls). Statistical significance was determined using Student's t-test, and p-values are shown for each protein. Proteins with $p < 0.05$ were considered significant DEPs.

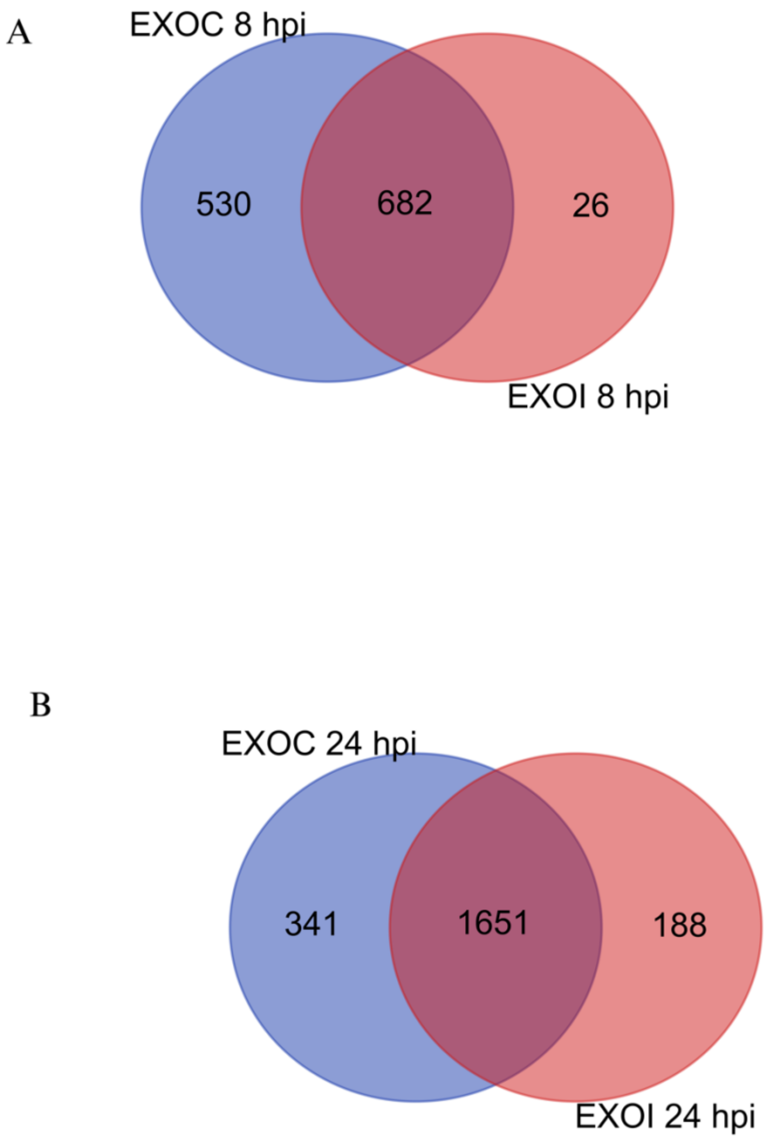

Supplementary Figure 1. Venn diagrams comparing the core proteomes of exosomes derived from uninfected macrophages (EXOC) and *B. abortus*–infected macrophages (EXOI). Only proteins consistently identified in all three biological replicates were considered. (A) At 8 hpi, 682 proteins were shared between conditions, while 530 were unique to EXOC and 26 were exclusive to EXOI. (B) At 24 hpi, 1,651 proteins were common to both conditions, with 341 unique to EXOC and 188 unique to EXOI.

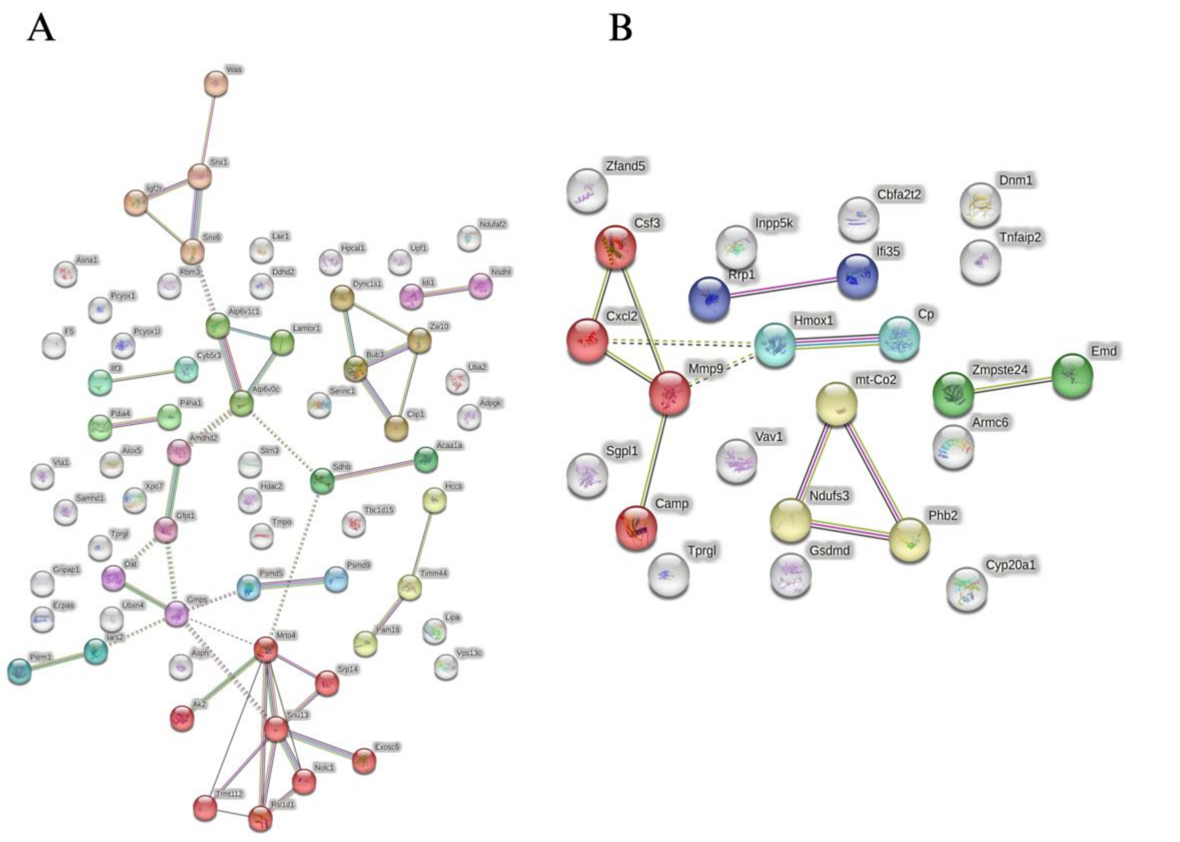

Supplementary Figure 2. Protein–protein interaction networks of proteins exclusively identified in exosomes derived from B. abortus–infected macrophages (EXOI). Networks were generated using STRING. (A) Exclusive proteins detected at 8 hpi. (B) Exclusive proteins detected at 24 hpi. All exclusive proteins are shown, including both interacting clusters and disconnected nodes without predicted STRING interactions.
